# Developing a process of lentivirus purification from recombinant fluids using peptide affinity ligands

**DOI:** 10.1002/btm2.70017

**Published:** 2025-04-07

**Authors:** Eduardo Barbieri, Gina N. Mollica, Sobhana A. Sripada, Shrirarjun Shastry, Yuxuan Wu, Arianna Minzoni, Will Smith, Elena Wuestenhagen, Annika Aldinger, Heiner Graalfs, Michael S. Crapanzano, Oliver Rammo, Michael M. Schulte, Michael A. Daniele, Stefano Menegatti

**Affiliations:** ^1^ Department of Chemical and Biomolecular Engineering North Carolina State University Raleigh North Carolina USA; ^2^ LigaTrap Technologies LLC Raleigh North Carolina USA; ^3^ Merck Life Sciences, KGaA Darmstadt Germany; ^4^ Joint Department of Biomedical Engineering North Carolina State University and University of North Carolina at Chapel Hill Raleigh North Carolina USA; ^5^ North Carolina Viral Vector Initiative in Research and Learning (NC‐VVIRAL) North Carolina State University Raleigh North Carolina USA; ^6^ Biomanufacturing Training and Education Center (BTEC) North Carolina State University Raleigh North Carolina USA

**Keywords:** affinity chromatography, lentivirus, membrane chromatography, peptide ligands, product polishing

## Abstract

Lentiviral vectors (LVVs) are emerging as an enabling tool in gene and cell therapies, yet the toolkit for purifying them at scale is still immature. A pivoting moment in LVV isolation technology was marked by the introduction of affinity ligands for LVVs pseudo‐typed with Vesicular Stomatitis Virus G (VSV‐G) protein. Camelid antibody ligands were initially discovered and utilized to functionalize a resin with a capacity of 10^14^ LVV particles per liter (vp/L). Shortly thereafter, our team introduced VSV‐G‐targeting peptides and assessed their application as ligands for purifying LVVs from HEK293 cell harvests. In this study, we utilized these peptides to develop novel affinity resins and—first in this field—affinity membranes with optimal binding capacity, productivity, and removal of host cell contaminants. To that end, we evaluated resins of different material, particle and pore size, and functional density, as well as membranes with different fiber morphology, porosity, and ligand distribution. The lead peptide‐functionalized resin and membrane featured high capacity (5 × 10^9^ and 1.2 × 10^9^ transducing LVV units per mL of adsorbent, TU/mL) and productivity (2.9 × 10^9^ and 1.7 × 10^9^ TU/mL min) and afforded a substantial enrichment of cell‐transducing LVVs and reduction of contaminants (110–170‐fold) in the eluates. Finally, we demonstrated an LVV purification process in four steps: clarification and nuclease treatment, affinity capture in bind‐and‐elute mode, polishing in flow‐through mode, and ultra/dia‐filtration and sterile filtration. The processes afforded yields of 33%–46%, a residual HCP level below 5 ng/mL, and productivity of 1.25–1.5 × 10^14^ active LVV particles per hour and liter of adsorbent.

## 1 INTRODUCTION

Modern gene and cell‐based therapies offer hope to patients with debilitating or life‐threatening diseases and aggressive forms of cancer.^1^ A milestone in cell therapy was reached in 2022, with the 10th anniversary of the first pediatric patient of acute lymphoblastic leukemia, Emily Whitehead, cured with Chimeric antigen receptor T (CarT) cell therapy.^2^ To date, the United States Food and Drug Administration (FDA) has approved more than 30 cell and gene therapies to fight a variety of conditions such as refractory multiple myeloma, B‐cell and follicular lymphoma, sickle cell disease, Hemophilia B, biallelic *RPE65* mutation‐associated retinal dystrophy, hematologic malignancies, congenital athymia, and β‐thalassemia.^3^

Several of these therapies rely on Lentiviral vectors (LVVs) for (*i*) the ex vivo modification of autologous—and in the future, potentially, heterologous—cells into Chimeric Antigen Receptor T cells (Car‐T) and natural killer (Car‐NK) cells; or (*ii*) the in vivo delivery of therapeutic genes for treating pediatric macular degeneration,^4^, ^5^, ^6^ beta‐thalassemia,^7^ or cerebral adrenoleukodystrophy.^8^ While endowed with outstanding therapeutic potential, LVVs are labile vectors—being sensitive to the physicochemical properties of the aqueous medium—and therefore challenging to manufacture affordably in clinically relevant amounts.^9^, ^10^, ^11^

Particularly critical in this context is the role of the purification technology, which is tasked with isolating cell‐transducing LVV particles from host cell proteins (HCPs), DNA (hcDNA), and extracellular vesicles, as well as the residual plasmids utilized for transient transfection and cell culture media.^11^, ^12^, ^13^, ^14^ To safeguard the therapeutic efficacy of LVVs and the safety of patients, the removal of contaminants must be conducted by minimizing the variations in temperature, pH, and osmotic pressure.

Affinity chromatography using ligands with bespoke biorecognition activity is ideal to this end. In 2023, the Peixoto's team developed affinity ligands targeting VSV‐G pseudotyped LVVs by screening a phage‐display library of single domain (V_H_H) camelid antibody fragments.^15^ The authors conjugated the protein ligands on agarose resins, achieving a binding capacity of ~10^11^ LVV particles per mL resin (vp/mL) at a 2 min residence time. The adsorbent was utilized to purify LVVs from HEK293 cell culture fluids, delivering values of vector recovery ranging from 45% to 54%, removal of HEK293 HCPs up to 99%, and hcDNA up to 80%. On the other hand, the lifetime, elution conditions, and stability to sodium hydroxide of the ligands require optimization.

In parallel, our team introduced a cohort of peptide ligands via combinatorial screening in tandem with in silico design.^16^ The lead peptides, conjugated on Poros™ resins, afforded LVV recovery of up to 60% and a ~200‐fold reduction of HCPs.^16^ Our initial study, however, did not optimize the parameters governing the purification performance of the peptide ligands, namely the material composition and morphology of the chromatographic matrix, and the ligand density and display. Accordingly, in the present study, we optimized the design of affinity adsorbents constructed using the lead peptides GKEAAFAA, FEKISNAE, and SRAFVGDADRD in combination with different matrices. Specifically, we sourced resins of different materials (i.e., polystyrene divinylbenzene, polymethyl methacrylate, polyvinyl ether, and agarose), diameter of the particles (45–90 μm) and of the pores (40–1000 nm), and functional density (0.02–0.1 mmol of peptide per mL of resin); we also tested membranes of different material (i.e., cellulose grafted with tentacle ligands^17^ and a polymeric fiber mat impregnated with a porous polyacrylamide hydrogel),^18^ pore diameter (~0.3–0.5 μm), and functional density (~0.05–0.2 mmol/mL). The resultant adsorbents varied widely in terms of LVV binding capacity as well as yield and purity. GKEAAFAA‐Poros™ resin, selected as the top‐performing adsorbent, was integrated into a 4‐step process of LVV purification that demonstrated the potential of our technology for large‐scale LVV manufacturing.

## 2 MATERIALS AND METHODS

### 2.1 Materials

Plasmids pALD‐LentiEGFP‐K, pALD‐Rev‐K, pALD‐VSV‐G‐K, and pALD‐GagPol‐K were purchased from Aldevron (Fargo, ND); dCAS9‐VP64_GFP was a gift from Feng Zhang (Addgene plasmid #61422)^19^; TransIT‐VirusGEN™ Transfection Reagent for LVV production was purchased from Mirus (Madison, WI). Viral production cells derived from HEK 293F, LV‐MAX production medium, LV‐MAX transfection kit, TrypLE™ express enzyme, fetal bovine serum (FBS), 5,5′‐Dithio‐bis‐(2‐nitrobenzoic acid), PureLink™ HiPure Plasmid Maxiprep Kit, Syto 13 dye, SulfoLink iodo activated resin, UltraLink iodoacetyl resin, Purelink Viral RNA/DNA Kit, 0.5 M Bond‐Breaker TCEP Solution, POROS™ 50 OH Hydroxyl Activated Resin, and high glucose DMEM supplemented with GlutaMAX™ and pyruvate were obtained from ThermoFisher Scientific (Waltham, MA). Trifluoroacetic acid (TFA), N,N′‐disuccinimidylcarbonate (DSC), 4‐dimethylaminopyridine (DMAP), Fmoc/tBu‐protected amino acids, piperidine, diisopropylethylamine (DIPEA), N‐Methyl‐2‐pyrrolidone (NMP), and hexafluorophosphate azabenzotriazole tetramethyl uronium (HATU) were purchased from Chem‐Impex (Wood Dale, Illinois). T‐75 and T‐25 cell culture flasks, 96‐well culture plates, DNAse/RNAse free water, BalanCD HEK293 medium, isopropanol, and ampicillin were sourced from VWR (Radnor, PA). HT1080 cell line was purchased from American Type Culture Collection (AATC) (Manassas, VA). N,N′‐Dimethylformamide (DMF), dichloromethane (DCM), sodium hydroxide, sodium chloride, sodium bicarbonate, 0.45 μm polyethersulfone (PES) vacuum filters, iodoacetyl chloride (IAC), triethylamine (TEA), acetonitrile (ACN), isopropanol (IPA), yeast extract, peptone, and granulated agar were obtained from Fisher Chemical (Hampton, NH). HIV1 p24 ELISA Kit was purchased from Abcam (Waltham, MA). HEK293 HCP ELISA kit was acquired from Cygnus (Southpoint, NC). ToyoPearl® amino‐750F and ToyoPearl® AF‐Amino‐650M were obtained from Tosoh bioscience (Tokyo, Japan). Epoxy‐activated Eshmuno® resins and membrane base materials for functionalization with GKEAAFAAC, SRAFVGDADRDC, and FEKISNAEC were donated by Merck Life Sciences KGaA (Darmstadt, Germany). Prepacked HiTrap Capto Core 700 columns, Peak Expression medium, and regenerated cellulose membranes with nominal pore size of 1 μm and 50‐mm thickness were purchased from Cytiva (Marlborough, MA). Transfection reagent PEIpro was purchased from Polyplus (Illkirch—France). Nalgene 0.2 μm syringe filters made of polyethersulfone (PES), Amicon ultra centrifugal filters (100 kDa), ethane‐1,2‐dithiol, 2‐mercaptoethanol, 1,4‐piperazinediethanesulfonic acid (PIPES) sesquisodium salt, branched polyethylenimine (molecular weight ~ 25,000 g/mol) (PEI), and benzonase were acquired from MilliporeSigma (Burlington, MA). Lyophilized peptides FEKISNAEC, GKEAAFAAC, and SRAFVGDADRDC, and iodoacetyl‐activated agarose resins were obtained from GenScript (Piscataway, NJ).

### 2.2 Resin functionalization with peptide ligands

The surface functionality of Poros™ resin and Eshmuno® 50 and 80 μm beads was converted to primary amino groups following the method described in prior work.^16^ Peptide sequences GKEAAFAA, GKEAAFAA‐G, GKEAAFAA‐GSG, GKEAAFAA‐GSGSGSG, GKEAAFAA‐GSGPGSG, GKEAAFAA‐PEG_3_, and FEKISNAE were synthesized on resins using an Initiator^+^ Alstra™ automated peptide synthesizer (Biotage, Uppsala, Sweden). Each amino acid coupling was performed by incubating a solution of 5 equivalents (eq.) of protected amino acid and HATU in dry DMF together with a solution of 6 eq. of DIPEA in NMP, both at concentrations of 0.5 M, with the resin for 20 min at 70°C (via microwave heating). After each coupling, Fmoc deprotection was conducted with 20% v/v piperidine in DMF for 30 min at room temperature. Final deprotection of the peptide chain was conducted using a cleavage cocktail containing TFA, thioanisole, anisole, and EDT (90/5/3/2 v/v) for 2 h at room temperature. Following deprotection, the resin was washed with DMF, DCM, dried with a stream of N_2_, and stored at 4°C. Bromide‐activated WorkBead resins were initially reacted with aqueous ammonia (25% v/v) at a 1:1 volume ratio at room temperature and under gentle mixing.^20^ After 16 h, 20 mL of aminated resin were washed with 10 volumes of water, 10 volumes of ethanol, and 10 volumes of acetonitrile, and subsequently incubated with a mixture composed of 0.717 mL of IAC, 1.112 mL of TEA, and 20 mL of ACN at room temperature under gentle mixing and in the dark. After 3 h, the resin was washed with 10 volumes of ACN, 10 volumes of acetone, and 10 volumes of DMF. The unreacted primary amines on the resin were acetylated by incubating 20 mL of resin with 13.2 mL of acetic anhydride and 20.4 mL of DIPEA in 40 mL of NMP for 3 h at room temperature. Completion of the reaction was confirmed via the Kaiser test. The conjugation of cysteine‐derivatized peptides on the iodoacetyl functionalized resins was conducted as described in prior work.^21^ Briefly, the resins were rinsed with water and 50 mM Tris, 5 mM EDTA‐Na, and 25 mM TCEP at pH 8.5 (coupling buffer). Peptides FEKISNAEC and GKEAAFAAC were dissolved in coupling buffer at 10 mg/mL and added to the resin at the ratio of 2 mL of peptide solution per mL of settled resin. The conjugation reaction was allowed to proceed at room temperature for 2 h under end‐to‐end mixing and was then quenched with 25 mM β‐mercaptoethanol in coupling buffer. The steps of resin activation, peptide conjugation, and quenching were conducted in the dark. The resin was thoroughly rinsed with 1 M sodium chloride and water and finally stored in 20% v/v ethanol at 4°C.

### 2.3 Amine activation of cellulose membranes

Regenerated cellulose membranes were initially rinsed with DMF and air dried. A membrane area of 100 cm^2^ was immersed in a solution composed of 35 mL of DMF, 1.80 g of DMAP, and 2.24 g of DSC for 3 h at room temperature and gentle agitation. The membranes were then rinsed with DMF, DMSO, and IPA, and kept in IPA at 4°C until the next step.^17^ The membranes were air dried and aminated by immersion in a solution composed of 10 g of PEI in 90 mL of MilliQ water at room temperature and gentle agitation. After 2 h, the membranes were rinsed with water, ACN, and functionalized with IAC following the procedure described in Section 2.2.

### 2.4 LVV production and harvest

The optimization of LVV expression by HEK293F cells is detailed in Section S1 (Figures S1 and S2), from which the following protocol was selected. Viral production cells (ThermoFisher Scientific, Waltham, MA) were initially cultured in BalanCD LV‐MAX or Peak Expression media at 8% CO_2_ and 37°C to reach a density of 3.5–5.5 × 10^6^ cells/mL for at least four passages before transfection. When using the PEIpro transfection reagent, the cells were diluted to 1.5 × 10^6^ cells/mL at 24 h before transfection and adjusted to 2.5 × 10^6^ cells/mL just before transfection. The PEIpro and plasmids were dissolved in DMEM media (10% of total cell culture volume) at a mass ratio of 1:3 (DNA:PEI) and 1 μg of total DNA per 10^6^ cells, mixed, and incubated for 15 min at room temperature before being added to the cell suspension.^15^ When using the LV‐Max system, the LVs were produced following the manufacturer's protocol.^22^ When using the Mirus transfection reagent, the plasmids were initially diluted in a volume of complex‐forming solution equal to 10% of the cell culture volume and added with a plasmid amount of 1.6 μg per every mL of cell culture suspension. Following plasmid dilution, a volume of transfection reagent solution at the ratio of 3 μL per every μg of plasmid was added to the same vial, mixed gently, and incubated for 15 min at room temperature before being added to the HEK293F cell suspension at 4.0 × 10^6^ cells/mL. The cell culture harvests were clarified via either centrifugation or depth filtration, each followed by microfiltration. The clarification via centrifugation was conducted as follows: the cells were removed after 48 h post transfection via centrifugation at 1300*g* for 15 min, the supernatants were treated with 50 U/mL of benzonase and 2 mM MgCl_2_ for 30 min at 37°C, and finally filtered using 0.45 μm polyethersulfone filters. The clarification via depth filtration was conducted following the procedure described by Mayani et al.^23^: the cell culture fluid (~10^7^  cells per mL at 85% viability) was loaded on a Millistak+® CE25 pod depth filter followed by a Millistak+® CE50 pod depth filter at the flux of 150 liters per square meter per hour, treated with 50 U/mL of benzonase and 2 mM MgCl_2_ for 30 min at 37°C, and finally filtered using a Polysep™ II cartridge filters (1.0/0.5 μm). The filters were flushed with 25 mM PIPES, 100 mM NaCl, pH 7.4 to increase LVV recovery. Unless immediately used, all samples were stored at −80°C.

### 2.5 LVV purification using peptide‐functionalized resins

Resins were flow packed into adjustable Tricorn 5/50 columns to a final volume of 1 mL and equilibrated with 10 column volumes (CVs) of binding buffer (25 mM PIPES, 100 mM NaCl, pH 7.4). A volume of 10–35 mL of clarified feedstock was loaded in down‐flow at the linear velocity of 305 cm/h (corresponding to a residence time (RT) of 1 min). Following resin wash with 20 CVs of binding buffer, LVV elution was conducted in up‐flow with 3 CVs of 25 mM PIPES, 650 mM NaCl, pH 7.4, and 3 CVs of 1 M NaCl, pH 7.4. Cleaning‐in‐Place (CIP) was conducted with 15 CVs of 0.5 M NaOH (aq) followed by static incubation for 15 min. The resin was finally washed with 10 CVs of equilibration buffer to restore neutral pH. All chromatographic steps were conducted at the flow rate of 1 mL/min (RT: 1 min), while continuously monitoring the conductivity, pH, and UV absorbance of the column effluents at 254, 260, and 280 nm. CaptureSelect™ Lenti VSVG affinity resin was operated following the manufacturer's instructions.^24^

### 2.6 Confocal Imaging of GKEAAFAA‐ *Poros™* after loading fluorescently tagged LVV particles

The lentiviruses were initially purified by sucrose gradient ultracentrifugation following the method described by Jiang et al.^25^ The resulting LVV pellet was suspended to a titer of ~1 × 10^11^ vp/mL in 0.5 mL of 25 mM PIPES, 100 mM NaCl, pH 7.4 overnight at 4°C. Fluorescent LVV labeling was conducted by incubating 2 μL of a solution of Syto 13 at 5 mM in DMSO with ~2 × 10^11^ LVV particles for 30 min at room temperature in the dark. The excess dye was removed using Pierce Dye Removal columns (ThermoFisher, MA). Fluorescently labeled LVVs were loaded onto GKEAAFAA‐Poros™ resin as described in Section 2.5. After loading, the beads were extracted from the front and back ends of the column and imaged using a Leica Stellaris Confocal Microscope (Wetzlar, Germany).

### 2.7 LVV purification using peptide‐functionalized membranes

Hydrogel‐filled membranes functionalized with peptides GKEAAFAAC, FEKISNAEC, and SRAFVGDADRDC were punched into disks of 22 mm in diameter. Two membrane layers were housed in a 25 mm Whatman filter holder (Cytiva, Marlborough, MA) and equilibrated with 50 membrane volumes (MVs) of 25 mM PIPES, 100 mM NaCl, pH 7.4 at 10 MV/min. A volume of 5 mL of clarified feedstock was loaded in down‐flow at 3 MV/min. After washing the membranes with 50 MVs of binding buffer, the LVV elution was conducted in up‐flow using 50 MVs of 25 mM PIPES, 650 mM NaCl, pH 7.4 at 10 MV/min. Finally, the membranes were regenerated with 50 MVs of 0.1 M glycine, 2 M NaCl, pH 2.0, and CIP was conducted with 50 MVs of 0.5 M NaOH (aq). Lentivirus purification with MustangQ devices (MV: 0.86 mL) was conducted following published work^26^: briefly, the membranes were initially equilibrated with 50 MVs of 10 mM histidine, 150 mM NaCl, pH 7.0; loaded with 150 MVs of clarified feedstock; and washed with 60 MVs of binding buffer. The LVV elution was performed in three steps using 20 MVs of 10 mM histidine buffer at pH 7.0 added with NaCl at concentrations of 0.4, 1.0, and 1.5 M, at the flow rate of 10 MV/min. All chromatographic steps were conducted while continuously monitoring the conductivity, pH, and UV absorbance of the column effluents at 254, 260, and 280 nm.

### 2.8 LVV polishing, buffer exchange, and sterile filtration

A 1 mL column packed with CaptoCore700 resin was equilibrated with 10 CVs of 25 mM PIPES, 100 mM NaCl, pH 7.4 at RT for 1 min and loaded in down‐flow with 20 CVs of the elution stream obtained from GKEAAFAA‐POROS™ (LVV titer: 6.2 × 10^9^ vp/mL; HCP titer: 2.6 μg/mL, Section 2.5) at RT for 2 min. The resin was cleaned in up‐flow with 15 CVs of 1 M NaOH in 30% (v/v) isopropanol: water followed by 30 min of static contact. All chromatographic steps were conducted while continuously monitoring the conductivity, pH, and UV absorbance of the column effluents at 254, 260, and 280 nm. After polishing, the LVVs were concentrated to a titer of 1.2 × 10^10^ vp/mL by centrifugation in Amicon filters (MWCO: 100 kDa) at 3500*g* for 30 min using 10 diavolumes of 25 mM PIPES, 10% sucrose, 20 mM MgCl_2_, pH 7.4. Finally, the samples were filtered using Nalgene PES 0.2 μm syringe filters and immediately analyzed or stored at −80°C.

### 2.9 Analysis of chromatographic samples

#### 2.9.1 Transduction assay

HT1080 cells were cultured in DMEM supplemented with 10% v/v FBS at 5% CO_2_ and 37°C until 80%–90% confluence was reached. Cells were released from the culture flasks using trypsin, counted using a hemocytometer and trypan blue for cell viability, and plated in a 96‐well plate at 7000 cells/mL. Plates were centrifuged at 900*g* for 5 min and kept in an incubator for 4 h. At the onset of the transduction assay, the culture media in the plates were replaced with equal volumes of samples prepared via serial dilution (10×) of the fractions containing LVVs (Sections 2.5, 2.7, and 2.8) in DMEM media supplemented with 8 μg/mL of polybrene. The affinity eluates obtained as described in Sections 2.5 and 2.7 were diluted and added to the cells within 15 min after collection to avoid a loss in LVV transduction activity. After 18 h, the spent medium was replaced with fresh DMEM medium supplemented with 10% v/v FBS, and the cells were incubated for 48 h. The fractions of cells expressing GFP were measured using a CytoFLEX flow cytometer (Beckman, Brea, CA) and the values of transduction units (TU) per mL were calculated using Equation (1). Only dilutions that yielded %GFP^+^ cells between 1% and 25% were considered for LVV transduction concentration.

(1) $$\text{Transduction}\;\left(\frac{\mathrm{TU}}{\mathrm{mL}}\right)=\frac{\text{number of cells}\times \left(\%\frac{{\mathrm{GFP}}^{+}}{100}\right)}{\text{volume}\;\left(\mathrm{mL}\right)\times \text{dilution factor}}$$

where the transduction units (TU) per mL were determined based on the number of cells at the time of transduction, the number of HT1080 cells expressing GFP, the total volume of sample per well, and the dilution factor.

#### 2.9.2 HEK293 HCP ELISA and p24 ELISA

The titer of p24 protein and HEK293 HCPs was respectively measured using HIV ELISA (Abcam, Waltham, MA) and HEK293 HCP ELISA (Cygnus, Southpoint, NC) kits following the manufacturer's instructions. From the values of HCP titer, the reduction values (RVs) and logarithmic reduction values (LRV) of HCPs were derived using Equation (2):

(2) RV=CHCP,ECAAV,ECHCP,LCAAV,LLRV=log10RV

where *C*_HCP,E_ and *C*_HCP,L_ are the HCP titers in the eluates and corresponding loads; *C*_LVV,E_ and *C*_LVV,L_ are the LVV titers in the eluates and corresponding loads.

#### 2.9.3 Real time quantitative PCR (RT‐qPCR)

RT‐qPCR was conducted as described in prior work.^16^ Briefly, DNAse‐treated samples were purified using a Purelink Viral RNA/DNA Kit (ThermoFisher Scientific, Waltham, MA) to isolate the encapsidated RNA. The samples were then combined with TaqMan fast virus, custom TaqMan probe, and the primers listed in Table S1, and analyzed using a CFX Duet Real‐Time qPCR System (Bio Rad, Hercules, CA). Plasmid pALD‐LentiEGFP‐K was used as a standard.

## 3 RESULTS

The growth of cell therapies as a transformative cure for severe diseases, including cancer and genetic disorders, calls for the introduction of an advanced bioprocessing toolkit for cell engineering and manufacturing.^27^ In this context, a key role is played by gene‐delivery vectors that can efficiently and safely transduce cells with a therapeutic transgene that confers a desired therapeutic functionality.^28^ To date, several gene‐delivery tools are available, including electroporation, gene guns, lipid nanoparticles (LNPs), and LVVs. Non‐viral delivery tools can be produced more easily than viral vectors and benefit from lower immunogenicity, but often face challenges such as lower transfection efficiency, shorter‐term gene expression, and higher potential for cell damage.^29^ Conversely, LVVs can deliver larger transgenes to both dividing and non‐dividing cells, enable stable gene integration, and have demonstrated clinical success.^30^ However, the first‐generation bioprocess toolkit for LVV design, expression, and purification is not suitable to meet the demand of modern cell therapies. Recent breakthroughs in viral vector design have significantly increased the precision and efficiency of gene delivery by LVVs, promoting their adoption in gene and cell therapies, particularly in oncology (e.g., CAR‐T cells).^31^, ^32^ Significant progress has also been made in recombinant LVV expression by increasing the titer, activity, and safety of LVVs expressed in mammalian cell lines.^33^ These innovations have catalyzed an increase in clinical trials that utilize LVVs,^34^ which rose globally from ~80 in 2014 (of which 20 in the United States) to over 370 in 2024 (~150 in the U.S.).^35^ Correspondingly, there has been a steady rise in regulatory approvals, with a total of eight FDA‐approved gene and cell therapies utilizing LVVs as of 2024.^36^ This upward trend has encouraged companies to invest in the research and development of LVVs, doubling the global market from US$ 127.6M in 2021 to US$ 292M in 2023—with projections indicating a market value of approximately US$ 1B by 2030, reflecting a compound annual growth rate (CAGR) of 18.5%.^37^

Nonetheless, several concerns persist regarding the costs of LVV production, which account for a substantial portion (~40%) of the total costs of cell therapy manufacturing.^35^, ^38^ These issues stem from the complexity and limited scalability of LVV bioprocessing. Recent improvements in upstream technology, such as the introduction of stable cell lines and perfusion bioreactors, have increased the productivity and efficiency of LVV expression. Consequently, the focus has shifted towards the purification pipeline, which accounts for 40%–60% of the total cost of goods in LVV production.^39^ Ideal purification tools must possess (*i*) high binding selectivity to isolate “mature” cell‐transducing LVVs—namely virions that comprise a transgene‐loaded capsid and an envelope that correctly displays pseudo‐typing proteins—from process‐related contaminants (e.g., host cell proteins and nucleic acids, the plasmids utilized for cell transfection, etc.) and product‐related impurities (e.g., immature virions); (*ii*) high binding capacity upon rapid loading and elution yield to maximize purification speed and productivity; and (*iii*) long lifetime and cost‐effectiveness to minimize overall manufacturing expenses. Addressing these needs, our team introduced affinity peptide ligands that target the VSV‐G protein utilized for pseudo‐typing the lentiviral envelope and demonstrated their ability to capture mature LVV particles and elute them under gentle conditions, delivering a highly pure and active product.^40^ Building on this foundation, the present study focuses on the design and operation of the peptide‐functionalized adsorbents towards optimizing the LVV binding capacity, yield and purity, and process productivity and lifetime. To ensure the comprehensiveness of our analysis, we compared resins and membranes of different material compositions and morphologies (i.e., particle and pore size, porosity, specific surface), and evaluated the role of conjugation chemistry, ligand display, and functional density. The selected peptide‐functionalized adsorbents demonstrated high binding capacity and product yield, achieving—in particular the membranes—high productivity, high product quality, and robust reusability.

### 3.1 Lentivirus purification performance of chromatographic resins functionalized with an affinity peptide ligand targeting VSV‐G

The material composition, porosity, specific surface area, and ligand density are critical design parameters that determine the binding capacity and selectivity of chromatographic adsorbents, and therefore the throughput and quality of the purified products.^41^, ^42^, ^43^, ^44^ Most of the published literature in this field focuses on the isolation of therapeutic proteins, especially monoclonal antibodies using affinity (e.g., Protein A, Protein G, and Protein L),^45^, ^46^ mixed‐mode,^47^, ^48^ and ion‐exchange ligands.^49^, ^50^ Yet, the impact of the above‐listed parameters on productivity and quality is arguably more pronounced with viral vectors, due to their larger size and complexity and lower stability.^51^, ^52^ Several studies investigated these phenomena in the context of the purification of adeno‐associated viral vectors (AAVs), focusing on either affinity adsorbents for product capture^53^, ^54^, ^55^, ^56^, ^57^, ^58^ or anion‐exchange for product polishing.^59^, ^60^, ^61^ Analogous studies on LVVs are, on the other hand, much less abundant despite the growing relevance of these vectors in cell and gene therapies.^11^ This is due partly to the difficult handling and analysis of LVVs and partly to the rather recent introduction of affinity ligands targeting LVVs pseudotyped with VSV‐G proteins. Seeking to fill this knowledge gap, this study focuses on the purification of LVVs using chromatographic substrates of different composition and morphology, beginning with resins made of polystyrene (Poros™), poly(methyl methacrylate) (ToyoPearl®), poly(vinylether) (Eshmuno®), polyacrylamide/azlactone (Ultralink), or crosslinked agarose (Genscript iodoacetyl and WorkBeads). The pore size of these matrices encompasses a broad range (50–10,000 nm), while bead diameter ranges from 45 to 75 μm.

We began our investigation by evaluating the role of substrate composition. In general, matrices constructed with biopolymers that are highly hydrophilic, which grant minimal non‐specific adsorption of host cell proteins and higher product purity; on the other hand, they are also characterized by a lower ligand density and consequently a lower binding capacity. In contrast, matrices made from synthetic polymers typically possess higher ligand density, and thus higher binding capacities, but tend to be more hydrophobic and—despite the surface of their pores being coated with a hydrophilic polymer layer (e.g., PEG)—adsorb more contaminants. The highest values of DBC_10%_ (1.9–5.0 × 10^9^ TU/mL of resin) were obtained with Eshmuno®, ToyoPearl®, and Poros™ resins, which are all synthetic polymer matrices and present the highest values of ligand density (0.1–0.15 mmol of peptide mL of resin, mmol/mL); in contrast, the capacity of agarose‐ and polyacrylamide/azlactone‐based resins, whose ligand density is more modest (0.02–0.05 mmol/mL), was consistently below 10^9^ TU/mL (Table 1; the complete set of design and performance parameters is presented in Table S2).

**TABLE 1:** Properties and performance of chromatography resins functionalized with peptide ligand GKEAAFAA.

Ligand	Resin	Ligand density (mmol/mL of resin)	DBC_10%_ (TU/mL of resin)	Productivity (TU/mL min)	HCP LRV
GKEAAFAA	ToyoPearl® 650	0.1	4.3 × 10^9^	2.6 × 10^9^	2.07
	ToyoPearl® 750	0.1	5.0 × 10^9^	2.4 × 10^9^	1.89
	Eshmuno® 50 μm	0.1	2.2 × 10^9^	1.2 × 10^9^	2.09
	Eshmuno® 80 μm	0.1	1.9 × 10^9^	1.1 × 10^9^	2.01
	Genscript iodoacetyl	0.02	5.6 × 10^8^	4.0 × 10^8^	2.24
	WorkBead	0.05	3.6 × 10^8^	1.8 × 10^8^	2.24
	SulfoLink	0.02	2.9 × 10^8^	1.6 × 10^8^	2.09
	Ultralink	0.025	4.3 × 10^8^	2.4 × 10^8^	1.75
	Poros™	0.1	4.5 × 10^9^	2.9 × 10^9^	2.04
Camelid antibody (V_H_H)	CaptureSelect™ Lenti VSVG Affinity Resin	Not disclosed	2.3 × 10^8^	0.7 × 10^8^ (RT: 2 min)	2.12

*Note*: The resins were packed in a 1 mL column, equilibrated with 100 mM NaCl in 25 mM PIPES buffer at pH 7.4, and loaded with clarified HEK293F cell culture fluid (LVV titer: 5–9 × 10^7^ TU/mL; HCP titer: 0.05 mg/mL) at the residence time (RT) of 1 min; following washing, LVV elution was conducted using 0.650 M NaCl in 25 mM PIPES at pH 7.4 at an RT of 1 min. Productivity was calculated as the number of cell‐transducing LVV units purified by 1 mL of resin in 1 min.

Additionally, the recovery of bound LVV particles appears to correlate to both particle and pore diameter. The efficiency of product release from a chromatographic resins decreases with the Thiele modulus, which is directly proportional to the kinetic constant of desorption and inversely proportional to the effective diffusion coefficient of the product and to the diameter of the porous particle. The effective diffusion coefficient, in turn, decreases with the hydrodynamic radius of the product and the tortuosity of the pores, while it increases with pore diameter. Accordingly, large targets such as viral vectors tend to show inefficient capture and release. This can be mitigated by reducing the Thiele modulus, which is achieved by using matrices with larger pores and smaller bead diameters. The results in Table 1 provide evidence to this claim. Firstly, Poros™ and ToyoPearl® 750 beads, which feature larger pores than the other resins, offered the highest LVV yield (60%–70%); similarly, ToyoPearl® 750 beads outperform ToyoPearl® 650 beads, despite having the same particle size, owing to their larger pore diameter. Finally, Eshmuno® 50 μm beads provide higher capacity and productivity than Eshmuno® 80 μm beads, despite having the same pore diameter, owing to their lower particle size. Based on the values of recovery, we derived the values of productivity, a key process‐relevant parameter defined as the number of cell‐transducing LVV units purified by 1 mL of resin in 1 min (TU/mL min). The values varied widely, ranging from as little as 2.4 × 10^8^ TU/mL min of GKEAAFAA‐Ultralink resin to as high as 2.9 × 10^9^ TU/mL min of GKEAAFAA‐Poros™ resin (41‐fold) higher than the commercial adsorbent CaptureSelect™ Lenti VSVG Affinity Resin.

Finally, all resins, with the exception of ToyoPearl® 750 and Ultralink, achieved a HCP reduction above 100‐fold. As anticipated, agarose‐based Genscript iodoacetyl and WorkBead resins, owing to their high hydrophilicity, afforded the highest clearance of host cell proteins (LRV ~ 2.24, corresponding to a 274‐fold reduction). In contrast, Eshmuno® and Poros™ resins afforded a lower impurity removal (LRV ~ 2, corresponding to a 100‐fold reduction), due in part to their relatively higher hydrophobicity. These results confirm our expectations that the LVV purification performance, while primarily driven by peptide GKEAAFAA, is also determined to a significant extent by the composition and morphological properties of the base matrix. Based on these results, GKEAAFAA‐Poros™ resin, whose binding capacity is ~2‐fold higher than the commercial VSV‐G Capture Select, was selected for further characterization.

### 3.2 Effect of gene on interest size on LVV purification

**FIGURE 1:**
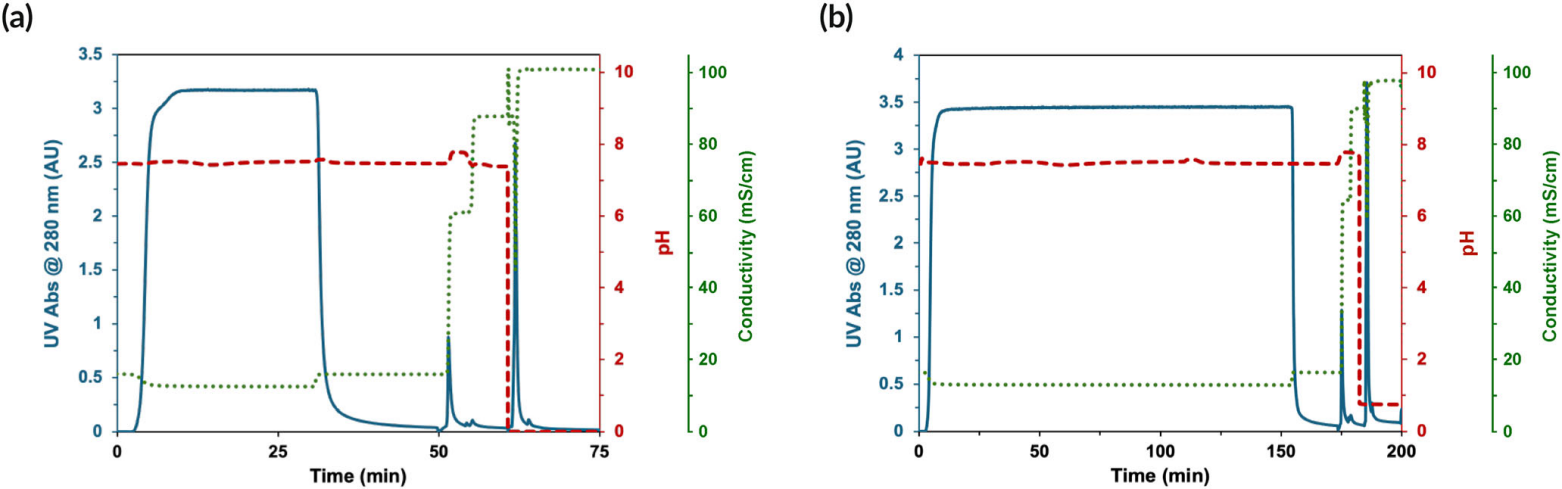
AKTA chromatograms obtained by loading clarified HEK293F cell culture fluids containing either (a) GFP‐LVV (LVV titer: 5 × 10^7^ TU/mL; HCP titer: 0.05 mg/mL) or (b) Cas9/GFP‐LVV (LVV titer: 2.02 × 10^6^ TU/mL; HCP titer: 0.05 mg/mL) on GKEAAFAA‐Poros™ resin. The resins were packed in a 1 mL column, equilibrated with 100 mM NaCl in 25 mM PIPES buffer at pH 7.4, and loaded with 150 mL of fluid at the RT of 1 min; following washing, LVV elution was conducted using 0.650 M NaCl in 25 mM PIPES at pH 7.4 at an RT of 1 min. The UV absorbance of the effluent was monitored at 280 nm.

Further characterization of GKEAAFAA‐Poros™ resin was conducted by evaluating the effect of GOI sequence and size. Although the size limit of the ssRNA cargo in LVVs is still debated,^62^ GOIs up to 10 kbases are considered the limit in terms of process feasibility and sufficient production of cell‐transducing vectors.^63^ Therefore, we evaluated the purification of LVVs packed with a GOI encoding for a fusion of CRISPR Cas9 nuclease and GFP (~9.5 kbases) from a clarified HEK293F cell culture fluid. As discussed in Section 3.1, increasing the GOI size leads to lower virus titer, requiring higher loading volume (Figure 1). The affinity purification afforded 40% virus recovery and a 4.5‐fold concentration factor, along with a 65‐fold reduction of HCPs to a residual titer of 4.14 μg/mL. The lower recovery can be attributed to the loading time, which increased from 30 min for GFP‐LVVs to 2.5 h for Cas9/GFP‐LVVs. Recent studies have shown that longer contact times of LVV with the chromatographic matrix lead to morphologic changes in LVVs and stronger multi‐point binding, reducing virus recovery.^64^

### 3.3 Effect of the spacer arm and ligand density on the performance of ligand GKEAAFAA

Introducing a spacer arm between the ligands and the chromatography support has been utilized since the dawn of affinity chromatography to promote product capture.^65^ Optimizing ligand display is particularly relevant in the context of viral vector purification, due to the large size and small curvature of the virion surface and the reliance on multi‐point interactions to achieve sufficient binding strength and capacity. We therefore evaluated the interposition of spacers of different composition, rigidity, and length—namely, a single amino acid G (gly), a tripeptide GSG (gly‐ser‐gly), two alternative heptapeptides GSGSGSG and GSGPGSG (gly‐ser‐gly‐ser‐gly‐ser‐gly and gly‐ser‐gly‐pro‐gly‐ser‐gly‐), and an oligoethylene glycol (PEG_3_)—between the C‐terminus of GKEAAFAA and the surface of POROS™ resin beads (Figure S3). The resulting adsorbents were loaded with clarified HEK293F cell culture fluid up to 75% of the anticipated binding capacity (DBC_10%_ ~ 5 × 10^9^ TU/mL of resin), at which point no virus was detected in either the flow‐through or the wash fractions. Somewhat unexpectedly, no significant differences in LVV recovery were recorded among the various adsorbents (Table 2).

**TABLE 2:** Values of recovery of cell‐transducing GFP‐LVV particles purified from a clarified HEK293F cell culture fluid (LVV titer: 7 × 10^7^ TU/mL; HCP titer: 0.05 mg/mL) using peptide ligand GKEAAFAA conjugated to Poros™ resin using different spacers.

Affinity resin	DBC_10%_ (TU/mL of resin)	Recovery of transducing LVV units	HCP LRV
GKEAAFAA‐Poros™	4.5 × 10^9^	65%	2.04
GKEAAFAA‐G‐Poros™	1.8 × 10^9^	42%	2.20
GKEAAFAA‐GSG‐Poros™	2.1 × 10^9^	62%	2.02
GKEAAFAA‐GSGPGSG‐Poros™	2.1 × 10^9^	41%	2.10
GKEAAFAA‐GSGSGSG‐Poros™	2.9 × 10^9^	54%	2.07
GKEAAFAA‐PEG_3_‐Poros™	2.8 × 10^9^	28%	2.08

*Note*: The resins were packed in a 1 mL column, equilibrated with 100 mM NaCl in 25 mM PIPES buffer at pH 7.4, and loaded with 40 mL of fluid at an RT of 1 min; following washing, LVV elution was conducted using 0.650 M NaCl in 25 mM PIPES at pH 7.4 at an RT of 1 min.

As mentioned above, the ligand density also determines the performance of chromatographic adsorbents, and its optimization is critical to strike an optimal balance between binding capacity and product yield^66^, ^67^: increasing the ligand density affords a higher binding capacity, but it can also reduce the binding selectivity as well as product recovery.^68^, ^69^ To evaluate the impact of ligand density on LVV binding capacity and productivity, we produced four lots of GKEAAFAA‐Poros™ resin ranging from 16 to 69 μmol/mL of resin (Table 3). Binding capacities did not vary substantially with ligand density, increasing by only 1.4‐fold against a 4‐fold difference in ligand density. Due to the large size of LVV particles, we expect the steric effect to be the primary factor determining capacity, while ligand density plays only a secondary effect, for an affinity resin of a given pore diameter. Conversely, tligand density determines the binding strength due to multi‐site interactions (i.e., avidity effect) and therefore product yield during elution. As described in a recent study by Bracewell et al.,^64^ the network of LVV:ligand interactions formed upon binding triggers the deformation of the virions on the chromatographic substrate from a spheroidal to a discoidal shape, resulting in even more bonds and ultimately leading to irreversible LVV adsorption. Indeed, we observed that the LVV recovery dropped from 80% to 37% as the ligand density increased, causing the productivity to decrease from 5 × 10^8^ to 3.3 × 10^8^ transducing units per mL of resin per minute (*note*: no LVV binding was recorded on the control OH‐Poros™ resin).

**TABLE 3:** Values of dynamic binding capacity (DBC_10%_) measured by p24 ELISA (viral particles per mL of resin, vp/mL), RT‐qPCR (viral transgenes per mL of resin, vg/mL), and transduction assay (transducing LVV units per mL of resin, TU/mL); recovery of LVV particles, transgenes, and transducing units; and HCP LRV obtained by purifying GFP‐LVV from a clarified HEK293F cell culture fluid (LVV particle titer: 1.7 × 10^10^ vp/mL; transgene titer: 3.9 × 10^8^ vg/mL; transducing units titer: 8 × 10^7^ TU/mL; HCP titer: 0.05 mg/mL) using peptide ligand GKEAAFAA conjugated to Poros™ resin with the ligand densities.

GKEAAFAA density (μmol/mL of resin)	DBC_10%_ (vp/mL)	DBC_10%_ (vg/mL)	DBC_10%_ (TU/mL)	Recovery of LVV particles	Recovery of LVV transgenes	Recovery of transducing LVV units	HCP LRV
68.5	5.4 × 10^10^	5.7 × 10^9^	5.3 × 10^9^	26%	44%	37%	1.94
34.2	4.4 × 10^10^	4.0 × 10^9^	3.9 × 10^9^	32%	66%	63%	2.04
28.1	4.2 × 10^10^	3.2 × 10^9^	4.4 × 10^9^	37%	78%	69%	1.93
15.9	4.0 × 10^10^	3.3 × 10^9^	3.7 × 10^9^	51%	81%	80%	1.99
0.0	—	—	<1 × 10^7^	—	—	<1%	—

*Note*: The resins were packed in a 1 mL column, equilibrated with 100 mM NaCl in 25 mM PIPES buffer at pH 7.4, and loaded with 40 mL of fluid at the RT of 1 min; following washing, LVV elution was conducted using 0.650 M NaCl in 25 mM PIPES at pH 7.4 at RT of 1 min.

### 3.4 Lifetime and stability study of GKEAAFAA‐Poros™ resin

The accessibility of gene and cell therapies relies on reducing the cost and increasing the sustainability of their manufacturing. Although most of the production costs are currently associated with upstream materials (e.g., culture media, plasmids, and transfection reagents), the development of stable cell lines for viral vector expression is likely to shift the focus of cost management to the downstream segment. In that context, the lifetime of chromatographic adsorbents is a critical factor in reducing operating costs and consumables waste streams. Affinity adsorbents in particular, due to their high cost, are expected to be reused over multiple cycles, each followed by a regeneration step using strong denaturing solvents and a cleaning‐in‐place (CIP) step using caustic conditions.^70^ The recommended CIP conditions for most of the affinity resins currently marketed for the purification of AAVs and LVVs are significantly milder (10 mM NaOH) than those routinely applied with established affinity adsorbents like Protein A/G resins (0.5 M NaOH).^24^

**FIGURE 2:**
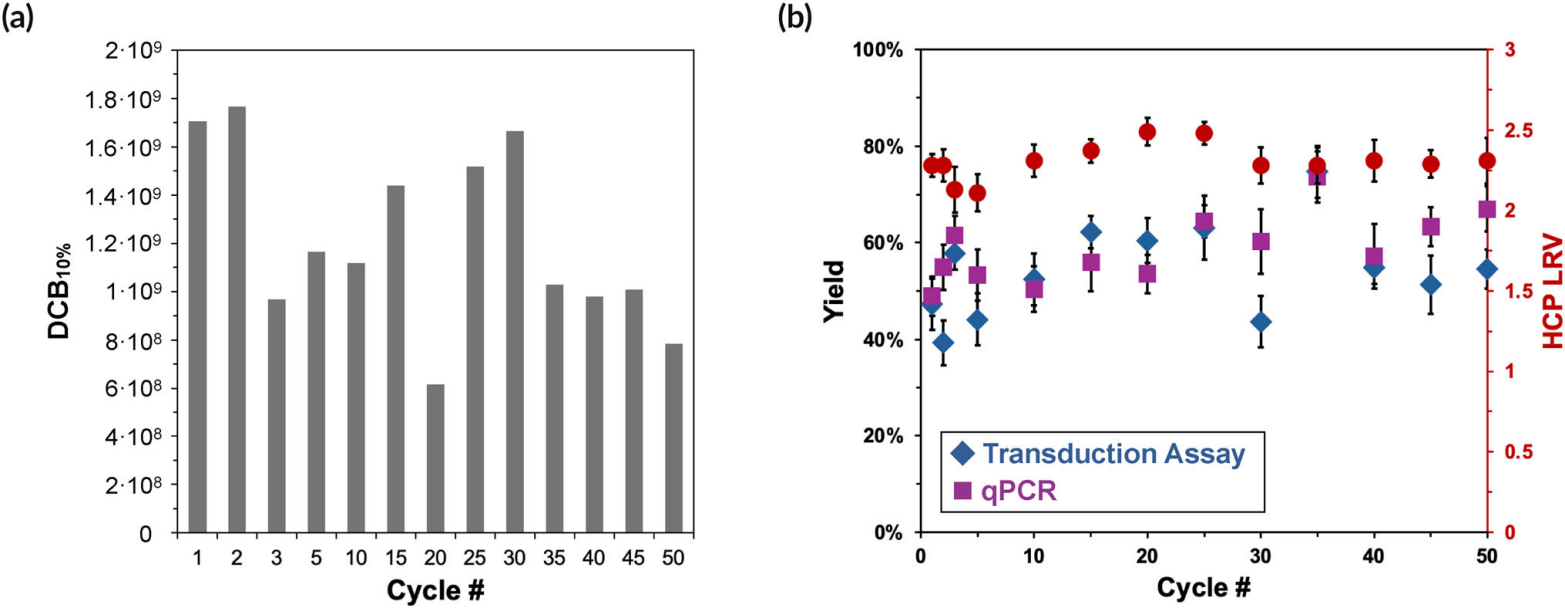
Caustic stability study of GKEAAFAA‐Poros™ resins conducted as consecutive cycles of LVV purification from the HEK293F cell culture fluid with intermediate CIP with 0.5 M NaOH (15 CVs at the RT of 1 min followed by 15 min of static contact time): (a) LVV binding capacity; (b) LVV elution yield determined by RT‐qPCR and transduction assay with HT1080 cells, and removal of HEK293 HCPs.

We therefore tested the ability of GKEAAFAA‐Poros™ to perform consecutive cycles of LVV purification with intermediate CIP using 0.5 M NaOH (15 CVs in flow followed by 15 min of static contact). The values of binding capacity, yield of encapsidated genomes and cell‐transducing LVV particles, and HCP removal measured over 50 cycles, summarized in Figure 2, demonstrate the stability of GKEAAFAA. This stems from the amino acid sequence of the peptide ligand, which does not contain residues prone to deamidation such as Asn (N) or Gln (Q),^71^, ^72^, ^73^, ^74^ or oxidation such as Trp (W), Cys (C), or Met (M).^75^, ^76^ The value of LVV DBC_10%_ fluctuated around 2 × 10^9^ TU per mL of resin, while the product yield and reduction of HEK293 HCP were consistently above 50% and 100‐fold. We note that the value of LVV Transducing Units (TU) depends not only on the lentiviral particle count but also on the average infectivity of the LVV batch and the viability of the HT1080 cells utilized in the transduction assay. Consequently, the value of DBC_10%_ in TU/mL is more subject to variability compared to the corresponding measure based on the physical titers of particles (vp/mL) or transgenes (vg/mL). For example, while the DBC_10%_ at cycle 20 was significantly lower (6 × 10^8^ TU/mL) than the average, the value of 1.5 × 10^9^ TU/mL was recorded at cycle 25. Nonetheless, reporting the DBC_10%_ in TU/mL better represents the affinity adsorbent's performance in purifying therapeutically active LVVs.

The desired lifetime of a chromatographic adsorbent depends upon its cost of goods, the value of the product being purified, and safety considerations (e.g., the risk of ligand leaching and the validation of column upon repacking). In the context of the proposed peptide‐functionalized resins and membranes, which are priced at US$ 12 K–14 K per liter—significantly lower than the costs of LVV expression (~US$ 225 K–275 K per dose)—the expected lifetime is 3–10 cycles. For comparison, the Protein A resins (US$ 12 K–18 K per liter) utilized for the purification of therapeutic monoclonal antibodies (US$ 50–100 per gram) have a much greater impact on bioprocessing cost, and their lifetime is therefore expected to reach 150–200 cycles. Since the proposed peptide‐functionalized adsorbents are reusable, it would be injudicious to re‐use a column for fewer than 3 cycles, because the time and resources required for column repacking and validation would become excessive. For instance, a standard 3‐Liter column packed with peptide‐functionalized adsorbents (DBC_10%_ ~ 1.2–4.5 × 10^9^ TU/mL) could process a 50‐Liter harvest at an LVV titer of 7 × 10^8^ TU/mL within 4–10 cycles. Having completely processed the harvest, the production campaign would terminate and the adsorbent would be replaced by default.

### 3.5 Membranes as an alternative substrate to chromatography resins for LVV purification

The high values of binding capacity characteristic of chromatography resins stem from the large surface area of their pores; however, the tortuous morphology and limited diameter of these pores reduce the transport of large biologics, such as viruses, due to diffusion limitations.^77^, ^78^, ^79^ Indeed, we confirmed that the binding of LVV particles is confined to the surface of the beads by loading fluorescently labeled LVVs on GKEAAFAA‐Poros™ and imaging the distribution of green fluorescence across the bead volume (Figure S4). Membranes offer an excellent alternative to resins as their open porosity eliminates diffusive limitations and enables processing at significantly higher flow rates and lower pressure drops.^80^ To date, however, most of the commercial affinity membranes are dedicated to protein purification,^81^, ^82^ whereas only a pseudo‐affinity sulfated cellulose membrane is used for purifying influenza A virions.^83^ Developing an affinity membrane would be particularly beneficial for LVV purification as it would safeguard the transduction activity of the purified virions by reducing their contact time with the chromatographic matrix.

**FIGURE 3:**
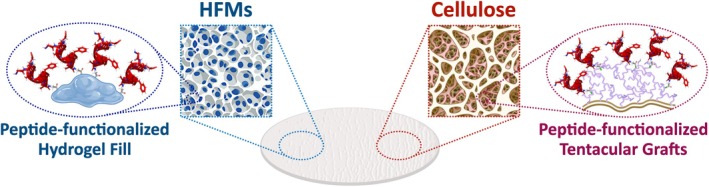
Structure of GKEAAFAA‐functionalized membranes: (left) hydrogel‐filled membranes (HFMs) functionalized with GKEAAFAA were prepared by functionalizing the hydrogel matrix loaded within the pore volume of the membrane scaffold; (right) GKEAAFAA‐cellulose membranes were prepared by functionalizing the branched polymer grafted on the surface of the pores.

Addressing these needs, we resolved to conjugate peptide ligands GKEAAFAA, FEKISNAE, and SRAFVGDADRD on prototype hydrogel‐filled membranes (HFMs) and activated cellulose membranes. HFMs comprise a polyamide fiber scaffold loaded with a continuous hydrogel matrix^18^ activated with cysteine‐reactive groups, which enable ligand conjugation at a functional density (~0.05 mmol per gram of membrane) comparable to that of resins. In parallel, we grafted a branched polyamine on the surface of cellulose membranes and used the resulting tentacular grafted layer for conjugating GKEAAFAA ligands at a comparable density (Figure 3). We evaluated the resulting adsorbents by measuring their DBC_10%_ at the residence time (RT) of 0.25 and 0.5 min, along with LVV recovery and HCP clearance (Table 4).

**TABLE 4:** Purification performance of affinity membranes functionalized with peptide ligands GKEAAFAA, FEKISNAE, and SRAFVGDADRD.

Membrane	RT (min)	DBC_10%_ (TU/mL)	Recovery of transducting LVVs (%)	Productivity (TU/mL min)	HCP LRV
FEKISNAE—HFM	0.25	(2.02 ± 0.21) × 10^8^	—		
	0.5	(3.05 ± 0.34) × 10^8^	29.5 ± 1.5%	1.8 × 10^8^	1.95 ± 0.11
GKEAAFAA—HFM	0.25	(3.15 ± 0.21) × 10^8^	—		
	0.5	(4.96 ± 0.37) × 10^8^	34.0 ± 3.0%	3.4 × 10^8^	2.18 ± 0.03
SRAFVGDADRD—HFM	0.5	(2.78 ± 0.03) × 10^8^	21.0 ± 0.5%	1.2 × 10^8^	2.69 ± 0.05
GKEAAFAA—cellulose	0.25	(2.30 ± 0.24) × 10^8^	87 ± 2.5%	6.4 × 10^8^	2.01 ± 0.09
	0.5	(1.23 ± 0.41) × 10^9^	74.0 ± 2.0%	1.7 × 10^9^	2.22 ± 0.12
Mustang Q	0.5	~10^10^	85.0 ± 0.5%	—	1.49 ± 0.05

*Note*: The membranes were packed in a 0.15 mL column, equilibrated with 100 mM NaCl in 25 mM PIPES buffer at pH 7.4, and loaded with clarified HEK293F cell culture fluid (LVV titer: 7 × 10^8^ TU/mL; HCP titer: 0.05 mg/mL) at the residence time (RT) of either 0.25 or 0.5 min; following washing, LVV elution was conducted using 0.650 M NaCl in 25 mM PIPES at pH 7.4 at an RT of 1 min. Productivity was calculated as the number of cell‐transducing LVV units purified by 1 mL of resin in 1 min.

The lower specific surface area—and thus the lower ligand density—resulted in a more modest binding capacity compared to GKEAAFAA‐Poros™ resin. However, it is important to note that GKEAAFAA‐HFM and GKEAAFAA‐cellulose membranes (pore size ~1 μm) afforded comparable productivity (~2 × 10^9^ TU/mL min) and purity (~220‐fold reduction of HCPs) by capitalizing on the short residence time during all steps of the chromatographic process. The difference in productivity resides in the lower recovery obtained with affinity membranes. This can be imputed to unfavorable steric effects in the polymer matrix surrounding or coating the fibers in the hydrogen‐filled membranes or, analogously, on the polycationic tentacles grafted on the cellulose membranes. These factors may indeed reduce the rate of LVV desorption, ultimately translating in lower recovery at short residence time. Notably, the functionalization strategy does not impair the binding selectivity of the affinity membranes, which consistently afforded highly purified LVVs. In particular, SRAFVGDADRD‐HFM afforded a remarkable ~500‐fold reduction of HCPs—to our knowledge, the highest value reported for LVV affinity purification. In comparison, Mustang Q (pore size ~0.8 μm), a strong anion‐exchange membrane widely utilized for LVV capture, afforded a higher product recovery but a significantly lower purity (i.e., 5‐fold lower reduction of HCPs than the affinity membranes).

We note that—to date—the effect of residual HCPs, hcDNA, and pDNA on engineered therapeutic cells has not been fully elucidated, and the only explicit recommendation issued by the FDA for LVV‐based ex vivo cell therapies is the clearance of replication‐competent LVVs. Process‐related contaminants, however, (*i*) pose significant risks of genomic instability, such as abnormal gene expression or oncogenesis, if the residual hcDNA contains active promoter regions or oncogenes; (*ii*) may compromise the efficacy of the therapeutic cells if the residual HCPs interfere with the function of the transduced cell; or (*iii*) may trigger an immune response in the recipient organism, potentially leading to inflammation or rejection of the transduced cells. As cell therapies advance, it is likely that the FDA will issue further guidelines regarding the removal of HCPs and hc/pDNA. In this context, affinity membranes such as GKEAAFAA‐cellulose are poised to become a tool of choice, given that they necessitate minimal post‐capture product polishing compared to alternatives such as Mustang Q.

### 3.6 Integrating peptide‐functionalized adsorbents in an LVV purification process

**FIGURE 4:**
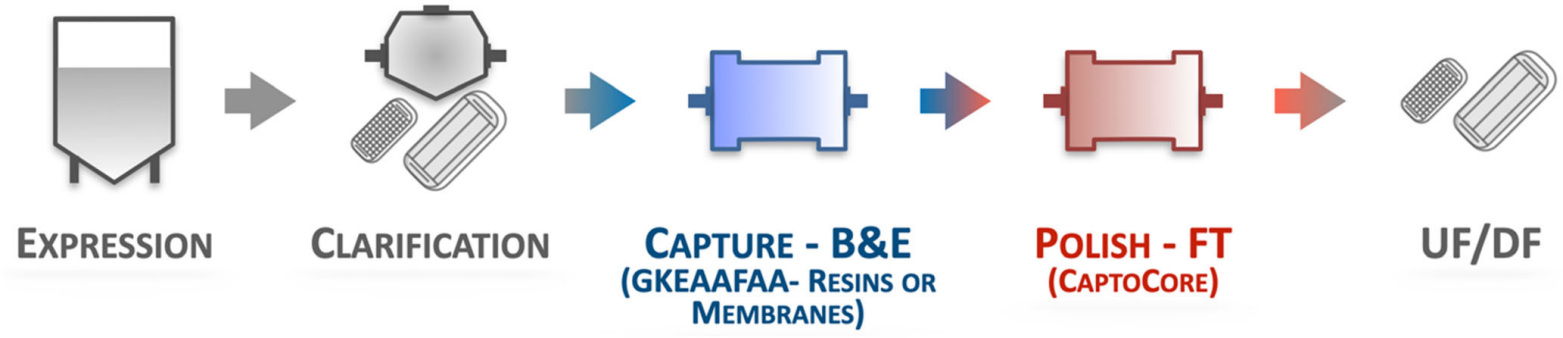
LVV manufacturing process comprising lentivirus expression via triple transfection of HEK293F cells, clarification via centrifugation and microfiltration, affinity‐based capture using either GKEAAFAA‐Poros™ resin or GKEAAFAA‐cellulose membranes in bind‐and‐elute mode, polishing in flow‐through mode, concentration via tangential flow filtration for concentration, and diafiltration and sterile filtration.

The results obtained in LVV expression and affinity purification coalesced in a downstream process comprising (*i*) clarification by centrifugation or depth filtration and microfiltration, (*ii*) affinity‐based capture in bind‐and‐elute mode using GKEAAFAA‐Poros™ resin or GKEAAFAA‐cellulose membranes, (*iii*) polishing in flow‐through mode, (*iv*) concentration via tangential flow filtration for concentration, and finally (*v*) diafiltration and sterile filtration (Figure 4). The HEK293F cell culture fluids were clarified and treated with benzonase to remove residual plasmids and host cell DNA (hcDNA). A negligible reduction in the titer of transducing LVV particles (~2%) was observed after centrifugation and filtration, whereas clarification by depth filtration caused a 39% loss of LVV transduction activity.

The clarified fluid was loaded onto the GKEAAFAA‐functionalized adsorbents to 100% of their binding capacity, since our prior observations in viral vector purification indicated that slightly overloading the column affords higher values of yield. LVV elution afforded a step LVV yield of 68% from GKEAAFAA‐Poros™ resin and 91% from GKEAAFAA‐cellulose membranes, corresponding to productivities of 1.25–2 × 10^14^ TU/h/L_resin_—together with a 150‐fold reduction in HCPs to a residual level below 0.5 μg/mL and residual DNA to an undetectable level. Most notably, the ratio of total versus infectious particles (TP/IP) decreased significantly at the affinity capture step from over 1500 to 145, corresponding to a remarkable 10‐fold enrichment in transducing LVVs.

Following the affinity step, a polishing step was implemented to remove residual impurities in flow‐through mode using CaptoCore700, which has been utilized in prior studies for the purification of large viral vectors (e.g., lentivirus and^84^ adenovirus). A CaptoCore bead comprises an outer layer (shell) whose pores exclude LVVs by size difference while allowing HCPs and other biomolecular contaminants to access the bead's inner, where they are captured by a combination of ion exchange and hydrophobic interactions. The polishing step afforded a high yield (87%–95%) and, together with concentration, an additional 100‐fold reduction in HCPs, achieving a residual level below 5 ng/mL (Table 5). The effluents from both processes were finally concentrated and buffer‐exchanged, and sterile‐filtered. While both processes provided a global 13,000‐fold reduction in HCP content and undetectable residual values of plasmid and host cell DNAs, the process employing the membrane‐based affinity adsorbent achieved a global yield of 46%, which is at the upper level of the range reported in the literature. These results corroborate the consensus in the bioprocess community, which is actively transitioning towards convective adsorbents for viral vector purification.

**TABLE 5:** Values of recovery, host cell protein removal, concentration factor, and total particles (TP) to infectious particles (IP) ratio obtained by purifying LVVs following the process in Figure 4 (clarification: Centrifugation and filtration; affinity capture: GKEAAFAA‐Poros™ resin).

Step	Step | Global recovery	Step | Global HCP LRV	Concentration factor	TP/IP ratio^a^
Filtration	98%	N/A	N/A	1531
Affinity capture (GKEAAFAA‐Poros™ resin)	68% | 67%	2.07	2.53	146
Polishing (CaptoCore 700)	95% | 64%	1.18 | 3.25	0.95	59
Concentration (TFF, MWCO: 100 kDa)	70% | 44%	0.86 | 4.11	5.16	30
Sterile filtration (0.2 μm filters)	75% | **33%**	N/A | **4.11**	N/A	**41 (37.3‐fold enrichment)**

*Note*: The bolded values represent the global recovery and purity, the concentration factor, and the ratio of active lentiviral particles at the end of the process.

^a^ Determined by p24 ELISA and transduction assay.

The key performance values reported in Tables 5 and 6 favors the membrane‐based process, which provides higher global recovery and purity as well as a better enrichment of cell‐transducing LVV units. Nonetheless, a global yield of ~33%–46%, while in line with reported values of LVV downstream, calls for an optimization of the various unit operations. In particular, significant product losses were located at the ultrafiltration/diafiltration and sterile filtration, which can be mitigated by optimizing the filters' material, microscopic morphology, and cut‐off values. Additional process development and scaling up efforts will focus on replacing the initial centrifugation with depth filtration for clarifying the feed and using flat membranes for ultrafiltration/diafiltration via TFF.

**TABLE 6:** Values of recovery, host cell protein removal, concentration factor, and total particles (TP) to infectious particles (IP) ratio obtained by purifying LVVs following the process in Figure 4 (clarification: Depth filtration and microfiltration; affinity capture: GKEAAFAA‐cellulose membranes).

Step	Step | Global recovery	Step | Global HCP LRV	Concentration factor	TP/IP ratio^a^
Filtration	98%	N/A	N/A	1531
Affinity capture (GKEAAFAA‐cellulose membranes)	91% | 89%	2.21	1.54	144
Polishing (CaptoCore 700)	87% | 78%	1.15 | 3.36	0.85	37
Concentration (TFF, MWCO: 100 kDa)	76% | 59%	0.77| 4.13	3.12	49
Sterile filtration (0.2 μm filters)	78% | **46%**	N/A | **4.13**	N/A	**51 (30.1‐fold enrichment)**

*Note*: The bolded values represent the global recovery and purity, the concentration factor, and the ratio of active lentiviral particles at the end of the process.

^a^ Determined by p24 ELISA and transduction assay.

## 4 CONCLUSIONS

The success of gene and cell therapies critically relies on the implementation of efficient, flexible, and robust bioprocess technologies tailored for viral vectors and engineered cells. The recent introduction of stable host cell lines, culture media for high product expression, and perfusion bioreactors that operate at high cell density has significantly improved the upstream segment of viral vector manufacturing, benefiting LVV production as well. Conversely, the technology offering for LVV purification is lagging, causing a bottleneck in the biomanufacturing of cell therapies. The bioprocessing community recognizes the urgent need for novel affinity adsorbents that feature high LVV‐binding selectivity and capacity at high flow rates, afford high product yield under mild elution conditions, and are robust and affordable. The anion‐exchange resins and membranes that are currently mainstream in LVV purification are affordable and feature high binding capacity, but their strong and poorly selective capture results in limited purity and recovery of active LVVs. In contrast, the CaptureSelect™ Lenti VSVG resin, which represents the only affinity adsorbent currently on the market, provides high binding selectivity but suffers from limited capacity, productivity, and reusability as well as high cost.

The peptide‐functionalized adsorbents developed in this work for the purification of VSV‐G‐pseudotyped LVVs feature (*i*) selective binding of mature, cell‐transducing LVV particles and gentle elution conditions,^40^ which deliver highly pure and active products that are free from host cell proteins and nucleic acid contaminants; and (*ii*) high binding capacity of LVVs carrying different genetic payloads at low residence times, which ensure high process flexibility and productivity. The combination of higher purification performance, longer lifetime, and lower cost of goods compared to commercial affinity technologies positions peptide‐functionalized adsorbents at the forefront of LVV purification technologies. For reference, the amount of affinity resin necessary to purify LVVs from a 3‐liter bioreactor, which supports the production of LVVs sufficient for a single cell therapy dose, is approximately 300 mL for CaptureSelect™ Lenti VSVG resin (price ~ $46 K per liter), 35 mL for GKEAAFAA‐Poros™ resin (~ $12 K per liter), and 75 mL of GKEAAFAA‐cellulose membrane (~ $14 K per liter).

Future studies will focus on increasing the yield of LVVs eluted from membranes by implementing strategies that accelerate the rate of desorption and increase the global yield of the LVV purification process beyond 50%. To this end, we will focus on optimizing fiber diameter and ligand density to increase the binding capacity and selectivity, while minimizing the thickness of the grafted layer for LVVs capture to increase product recovery. We also anticipate formulating the elution buffer using kosmotropic salts and additives that further improve product yield and stability. We will then integrate the optimized affinity step with efficient pre‐ and post‐chromatographic filters, resulting in a streamlined purification process that delivers clinically relevant quantities of LVVs at a fraction of the current manufacturing costs, thus expanding access for patients to LVV‐based cell therapies.

## AUTHOR CONTRIBUTIONS

**Eduardo Barbieri:** Conceptualization; investigation; writing – original draft; methodology; data curation. **Gina N. Mollica:** Investigation. **Sobhana A. Sripada:** Investigation. **Shrirarjun Shastry:** Investigation. **Yuxuan Wu:** Investigation. **Arianna Minzoni:** Investigation. **Will Smith:** Investigation. **Elena Wuestenhagen:** Conceptualization; investigation; writing – review and editing. **Annika Aldinger:** Conceptualization; investigation; writing – review and editing. **Heiner Graalfs:** Conceptualization; investigation; writing – review and editing. **Michael S. Crapanzano:** Conceptualization; investigation; funding acquisition; writing – review and editing; supervision. **Oliver Rammo:** Conceptualization; writing – review and editing; investigation. **Michael M. Schulte:** Conceptualization; investigation; writing – review and editing; supervision. **Michael A. Daniele:** Funding acquisition; writing – review and editing; supervision. **Stefano Menegatti:** Conceptualization; investigation; funding acquisition; writing – original draft; writing – review and editing; methodology; data curation; supervision.

## CONFLICT OF INTEREST STATEMENT

The authors declare no conflict of interest.

## Supporting information


**APPENDIX S1:** Supporting information.

## Data Availability

The data that support the findings of this study are available from the corresponding author upon reasonable request.

## References

[1] U.S. Food and Drug Admistration . Rare Diseases at FDA. 2022 https://www.fda.gov/patients/rare-diseases-fda

[2] Grupp SA , Kalos M , Barrett D , et al. Chimeric antigen receptor‐modified T cells for acute lymphoid leukemia. N Engl J Med. 2013;368:1509‐1527. 23527958 10.1056/NEJMoa1215134 PMC4058440

[3] United States Food and Drug Administration . Approved Cellular and Gene Therapy Products. 2023 https://www.fda.gov/vaccines‐blood‐biologics/cellular‐gene‐therapy‐products/approved‐cellular‐and‐gene‐therapy‐products

[4] Campochiaro PA , Lauer AK , Sohn EH , et al. Lentiviral vector gene transfer of endostatin/angiostatin for macular degeneration (GEM) study. Hum Gene Ther. 2016;28:99‐111. 27710144 10.1089/hum.2016.117 PMC5278797

[5] Balaggan KS , Ali RR . Ocular gene delivery using lentiviral vectors. Gene Ther. 2012;19:145‐153. 22052240 10.1038/gt.2011.153

[6] Arsenijevic Y , Berger A , Udry F , Kostic C . Lentiviral vectors for ocular gene therapy. Pharmaceutics. 2022;14:1605. 36015231 10.3390/pharmaceutics14081605 PMC9414879

[7] U.S. Food and Drug Administration, C. for D. E. and R . STN: BL 125717/0, Approval Letter. 2022 https://www.fda.gov/media/160994/download?attachment

[8] U.S. Food and Drug Administration, C. for D. E. and R . BL 125755/34 Supplement Approval. 2024.

[9] Martínez‐Molina E , Chocarro‐Wrona C , Martínez‐Moreno D , Marchal JA , Boulaiz H . Pharmaceutics large‐scale production of lentiviral vectors: current perspectives and challenges. Pharmaceutics. 2020;12:1051. 33153183 10.3390/pharmaceutics12111051 PMC7693937

[10] Shupe J , Zhang A , Odenwelder DC , Dobrowsky T . Gene therapy: challenges in cell culture scale‐up. Curr Opin Biotechnol. 2022;75:102721. 35398708 10.1016/j.copbio.2022.102721

[11] Moreira AS , Cavaco DG , Faria TQ , Alves PM , Carrondo MJT , Peixoto C . Advances in lentivirus purification. Biotechnol J. 2021;16(1):2000019. doi:10.1002/biot.202000019 33089626

[12] Johnson S , Wheeler JX , Thorpe R , Collins M , Takeuchi Y , Zhao Y . Mass spectrometry analysis reveals differences in the host cell protein species found in pseudotyped lentiviral vectors. Biologicals. 2018;52:59‐66. 29361371 10.1016/j.biologicals.2017.12.005 PMC5910304

[13] Do Minh A , Star AT , Stupak J , et al. Characterization of extracellular vesicles secreted in lentiviral producing HEK293SF cell cultures. Viruses. 2021;13:797. 33946875 10.3390/v13050797 PMC8145507

[14] Ausubel LJ , Hall C , Sharma A , et al. Production of CGMP‐grade lentiviral vectors. Bioprocess Int. 2012;10:32‐43. 22707919 PMC3374843

[15] Moreira AS , Bezemer S , Faria TQ , et al. Implementation of novel affinity ligand for lentiviral vector purification. Int J Mol Sci. 2023;24:3354. 36834764 10.3390/ijms24043354 PMC9966744

[16] Barbieri E , Mollica GN , Moore BD , et al. Peptide ligands targeting the vesicular stomatitis virus G (VSV‐G) protein for the affinity purification of lentivirus particles. Biotechnol Bioeng. 2024;121:618‐639. 37947118 10.1002/bit.28594

[17] Edioma F . Development of a new affinity membrane for rapidly purifying non‐antibody proteins. (Clemson University, Clemson, SC). 2022.

[18] Hou Y , Brower M , Pollard D , et al. Advective hydrogel membrane chromatography for monoclonal antibody purification in bioprocessing. AIChE Biotechnol Prog. 2015;31:974‐982. 10.1002/btpr.2113 26018631

[19] Konermann S , Brigham MD , Trevino AE , et al. Genome‐scale transcriptional activation by an engineered CRISPR‐Cas9 complex. Nature. 2015;517(7536):583‐588. doi:10.1038/nature14136 25494202 PMC4420636

[20] Kish WS , Roach MK , Sachi H , Naik AD , Menegatti S , Carbonell RG . Purification of human erythropoietin by affinity chromatography using cyclic peptide ligands. J Chromatogr B. 2018;1085:1‐12. 10.1016/j.jchromb.2018.03.039 29625371

[21] Domen PL , Nevens JR , Mallia AK , Hermanson GT , Klenk DC . Site‐directed immobilization of proteins. J Chromatogr A. 1990;510:293‐302. 10.1016/s0021-9673(01)93763-x 2401701

[22] Gibco . LV‐MAX™ Lentiviral Production System—User Guide. 2021 https://assets.thermofisher.com/TFS-Assets/LSG/manuals/MAN0017000_LV_MAX_ViralProductionSystem_UG.pdf

[23] Mayani M , Nellimarla S , Mangalathillam R , et al. Depth filtration for clarification of intensified lentiviral vector suspension cell culture. Biotechnol Prog. 2024;40:e3409. 37985144 10.1002/btpr.3409

[24] Fisher Scientific T . CaptureSelect Lenti VSVG Affinity Matrix Product Information Sheet (Pub. No. MAN0028036 A.0). www.thermofisher.com/captureselect

[25] Jiang W , Hua R , Wei M , et al. An optimized method for high‐titer lentivirus preparations without ultracentrifugation. Sci Rep. 2015;5:13875. 26348152 10.1038/srep13875 PMC4562269

[26] Moreira AS , Faria TQ , Oliveira JG , et al. Enhancing the purification of lentiviral vectors for clinical applications. Sep Purif Technol. 2021;274:118598.

[27] El‐Kadiry AEH , Rafei M , Shammaa R . Cell therapy: types, regulation, and clinical benefits. Front Med Lausanne. 2021;8: 1‐24. 10.3389/fmed.2021.756029 PMC8645794 34881261

[28] Taghdiri M , Mussolino C . Viral and non‐viral systems to deliver gene therapeutics to clinical targets. Int J Mol Sci. 2024;25(13):7333. doi:10.3390/ijms25137333 39000440 PMC11242246

[29] Shahlaei M , Asl SM , Saeidifar M . Nanotechnology in gene delivery for neural regenerative medicine. Neural Regener Nanomed. 2020;123‐157.

[30] Butt MH , Zaman M , Ahmad A , et al. Appraisal for the potential of viral and nonviral vectors in gene therapy: a review. Genes Basel. 2022;13(8):1370. doi:10.3390/genes13081370 36011281 PMC9407213

[31] Segura MM , Mangion M , Gaillet B , Garnier A . New developments in lentiviral vector design, production and purification. Expert Opin Biol Ther. 2013;13:987‐1011. 23590247 10.1517/14712598.2013.779249

[32] Dong W , Kantor B . Lentiviral vectors for delivery of gene‐editing systems based on CRISPR/Cas: current state and perspectives. Viruses. 2021;13(7):1288. doi:10.3390/v13071288 34372494 PMC8310029

[33] Ferreira MV , Cabral ET , Coroadinha AS . Progress and perspectives in the development of lentiviral vector producer cells. Biotechnol J. 2021;16:2000017. 10.1002/biot.202000017 32686901

[34] Milone MC , O'Doherty U . Clinical use of lentiviral vectors. Leukemia. 2018;32:1529‐1541. 29654266 10.1038/s41375-018-0106-0 PMC6035154

[35] Jadlowsky JK , Leskowitz R , McKenna S , et al. Long‐term stability of clinical‐grade lentiviral vectors for cell therapy. Mol Ther Methods Clin Dev. 2024;32:101186. 38282894 10.1016/j.omtm.2024.101186 PMC10811425

[36] U.S. Food & Drug Administration . Approved cellular and gene therapy products. 2024 https://www.fda.gov/vaccines-blood-biologics/cellular-gene-therapy-products/approved-cellular-and-gene-therapy-products

[37] Coherent Market Insights . Lentiviral vectors market analysis, 1–220. 2023 https://www.coherentmarketinsights.com/market-insight/lentiviral-vectors-market-4068

[38] Comisel RM , Kara B , Fiesser FH , Farid SS . Lentiviral vector bioprocess economics for cell and gene therapy commercialization. Biochem Eng J. 2021;167:107868.

[39] Merten OW , Hebben M , Bovolenta C . Production of lentiviral vectors. Mol Ther Methods Clin Dev. 2016;3:16017. 27110581 10.1038/mtm.2016.17 PMC4830361

[40] Sripada SA , Barbieri E , Shastry S , et al. Multiangle light scattering as a lentivirus purification process analytical technology. Anal Chem. 2024;96:9593‐9600. 38804040 10.1021/acs.analchem.4c01209

[41] Sánchez‐Trasviña C , Flores‐Gatica M , Enriquez‐Ochoa D , Rito‐Palomares M , Mayolo‐Deloisa K . Purification of modified therapeutic proteins available on the market: an analysis of chromatography‐based strategies. Front Bioeng Biotechnol. 2021;9:717326. 34490225 10.3389/fbioe.2021.717326 PMC8417561

[42] Roberts JA , Kimerer L , Carta G . Effects of molecule size and resin structure on protein adsorption on multimodal anion exchange chromatography media. J Chromatogr A. 2020;1628:461444. 32822983 10.1016/j.chroma.2020.461444

[43] Bagge J , Enmark M , Leśko M , Limé F , Fornstedt T , Samuelsson J . Impact of stationary‐phase pore size on chromatographic performance using oligonucleotide separation as a model. J Chromatogr A. 2020;1634:461653. 33171435 10.1016/j.chroma.2020.461653

[44] Trilisky EI , Lenhoff AM . Flow‐dependent entrapment of large bioparticles in porous process media. Biotechnol Bioeng. 2009;104:127‐133. 19459138 10.1002/bit.22370 PMC2782472

[45] Sun YN , Shi C , Zhang QL , et al. Comparison of protein A affinity resins for twin‐column continuous capture processes: process performance and resin characteristics. J Chromatogr A. 2021;1654:462454. 34407469 10.1016/j.chroma.2021.462454

[46] Swinnen K , Krul A , van Goidsenhoven I , van Tichelt N , Roosen A , van Houdt K . Performance comparison of protein A affinity resins for the purification of monoclonal antibodies. J Chromatogr B. 2007;848:97‐107. 10.1016/j.jchromb.2006.04.050 16765655

[47] O'Connor E , Aspelund M , Bartnik F , et al. Monoclonal antibody fragment removal mediated by mixed mode resins. J Chromatogr A. 2017;1499:65‐77. doi:10.1016/j.chroma.2017.03.063 28389094

[48] Koehnlein W , Holzgreve A , Schwendner K , Skudas R , Schelter F . Purification of hydrophobic complex antibody formats using a moderately hydrophobic mixed mode cation exchange resin. J Chromatogr A. 2023;1687:463696. 36508767 10.1016/j.chroma.2022.463696

[49] Roshankhah R , Chen G , Xu Y , et al. Purification of monoclonal antibody using cation exchange z2 laterally‐fed membrane chromatography – a potential alternative to protein A affinity chromatography. Biochem Eng J. 2022;178:108293.

[50] Fan J , Sripada SA , Pham DN , et al. Purification of a monoclonal antibody using a novel high‐capacity multimodal cation exchange nonwoven membrane. Sep Purif Technol. 2023;317:123920.

[51] Singh N , Heldt CL . Challenges in downstream purification of gene therapy viral vectors. Curr Opin Chem Eng. 2022;35:100780.

[52] Lothert K , Harsy YMJ , Endres P , Müller E , Wolff MW . Evaluation of restricted access media for the purification of cell culture‐derived Orf viruses. Eng Life Sci. 2023;23:e2300009. 37664009 10.1002/elsc.202300009 PMC10472920

[53] Fan J , Barbieri E , Shastry S , Menegatti S , Boi C , Carbonell RG . Membranes purification of adeno‐associated virus (AAV) serotype 2 from Spodoptera frugiperda (Sf9) lysate by chromatographic nonwoven membranes. Membranes. 2022;12:944. 36295703 10.3390/membranes12100944 PMC9606886

[54] Chu W , Shastry S , Barbieri E , et al. Peptide ligands for the affinity purification of adeno‐associated viruses from HEK 293 cell lysates. Biotechnol Bioeng. 2023;120:2283‐2300. 37435968 10.1002/bit.28495 PMC10440015

[55] Pulicherla N , Asokan A . Peptide affinity reagents for AAV capsid recognition and purification. Gene Ther. 2011;18:1020‐1024. 21490687 10.1038/gt.2011.46 PMC3192935

[56] Mietzsch M , Smith JK , Yu JC , et al. Characterization of AAV‐specific affinity ligands: consequences for vector purification and development strategies. Mol Ther Methods Clin Dev. 2020;19:362‐373. 33145372 10.1016/j.omtm.2020.10.001 PMC7591348

[57] Florea M , Nicolaou F , Pacouret S , et al. High‐efficiency purification of divergent AAV serotypes using AAVX affinity chromatography. Mol Ther Methods Clin Dev. 2023;28:146‐159. 36654797 10.1016/j.omtm.2022.12.009 PMC9823220

[58] Heckel J , Martinez A , Elger C , et al. Fast HPLC‐based affinity method to determine capsid titer and full/empty ratio of adeno‐associated viral vectors. Mol Ther Methods Clin Dev. 2023;31:101148. 38046198 10.1016/j.omtm.2023.101148 PMC10690635

[59] Kurth S , Li T , Hausker A , et al. Separation of full and empty adeno‐associated virus capsids by anion‐exchange chromatography using choline‐type salts. Anal Biochem. 2024;686:115421. 38061416 10.1016/j.ab.2023.115421

[60] Di W , Koczera K , Zhang P , Chen DP , Warren JC , Huang C . Improved adeno‐associated virus empty and full capsid separation using weak partitioning multi‐column AEX chromatography. Biotechnol J. 2024;19:2300235. 10.1002/biot.202300245 38013662

[61] Lavoie RA , Zugates JT , Cheeseman AT , et al. Enrichment of adeno‐associated virus serotype 5 full capsids by anion exchange chromatography with dual salt elution gradients. Biotechnol Bioeng. 2023;120:2953‐2968. 37256741 10.1002/bit.28453

[62] Kumar M , Keller B , Makalou N , Sutton RE . Systematic determination of the packaging limit of lentiviral vectors. Hum Gene Ther. 2001;12:1893‐1905. 11589831 10.1089/104303401753153947

[63] Kalidasan V , Ng WH , Ishola OA , Ravichantar N , Tan JJ , das KT . A guide in lentiviral vector production for hard‐to‐transfect cells, using cardiac‐derived c‐kit expressing cells as a model system. Sci Rep. 2021;11:19265. 34584147 10.1038/s41598-021-98657-7 PMC8478948

[64] Pamenter G , Davies L , Knevelman C , et al. Time‐dependent sorption behavior of lentiviral vectors during anion‐exchange chromatography. Biotechnol Bioeng. 2023;120:2269‐2282. 37386920 10.1002/bit.28483

[65] DePhillips P , Lagerlund I , Färenmark J , Lenhoff AM . Effect of spacer arm length on protein retention on a strong cation exchange adsorbent. Anal Chem. 2004;76:5816‐5822. 15456302 10.1021/ac049462b

[66] Černigoj U , Vidic U , Nemec B , et al. Characterization of methacrylate chromatographic monoliths bearing affinity ligands. J Chromatogr A. 2016;1464:72‐78. 27554023 10.1016/j.chroma.2016.08.014

[67] Zhao WW , Shi QH , Sun Y . FYWHCLDE‐based affinity chromatography of IgG: effect of ligand density and purifications of human IgG and monoclonal antibody. J Chromatogr A. 2014;1355:107‐114. 24947889 10.1016/j.chroma.2014.05.083

[68] Nelson SL , Li Y , Chen Y , Deshmukh L . Avidity‐based method for the efficient generation of monoubiquitinated recombinant proteins. J Am Chem Soc. 2023;145:7748‐7752. 37010382 10.1021/jacs.3c01943 PMC10103170

[69] Ollier R , Wassmann P , Monney T , et al. mAbs single‐step protein A and protein G avidity purification methods to support bispecific antibody discovery and development single‐step protein A and protein G avidity purification methods to support bispecific antibody discovery and development. MAbs. 2019;11:1464‐1478. 31462177 10.1080/19420862.2019.1660564 PMC6816383

[70] Mayela Ramos‐de‐la‐Peña José González‐Valdez Oscar Aguilar A , Mayela Ramos‐de‐la‐Peña A , Aguilar O , Garza Sada E . Methods chromatography · electroseparation applications biomedicine · foods · environment protein A chromatography: challenges and progress in the purification of monoclonal antibodies. JSSCCJ. 2019;42:1659‐1828. 10.1002/jssc.201800963 30811843

[71] Robinson NE . Protein deamidation. PNAS. 2022;99:5283‐5288. 10.1073/pnas.082102799 PMC122761 11959979

[72] Patel K , Borchardt RT . Chemical pathways of peptide degradation. II. Kinetics of deamidation of an asparaginyl residue in a model hexapeptide. Pharm Res. 1990;7:703‐711. 2395797 10.1023/a:1015807303766

[73] Lura R , Schirch V . Role of peptide conformation in the rate and mechanism of deamidation of asparaginyl residues. Biochemistry. 1988;27:7671‐7677. 3207697 10.1021/bi00420a015

[74] Kosky AA , Razzaq UO , Treuheit MJ , Brems DN . The effects of alpha‐helix on the stability of Asn residues: deamidation rates in peptides of varying helicity. Protein Sci. 1999;8:2519‐2523. 10595558 10.1110/ps.8.11.2519 PMC2144212

[75] Yang Y . Peptide oxidation/reduction side reactions. Side React Peptide Synth. 2016;3:43‐75.

[76] Fuentes‐Lemus E , Dorta E , Escobar E , et al. Oxidation of free, peptide and protein tryptophan residues mediated by AAPH‐derived free radicals: role of alkoxyl and peroxyl radicals. RSC Adv. 2016;6:57948‐57955.

[77] Do Minh A , Kamen AA . Critical assessment of purification and analytical technologies for enveloped viral vector and vaccine processing and their current limitations in resolving co‐expressed extracellular vesicles. Vaccine. 2021;9(823): 1‐16. 10.3390/vaccines9080823 PMC8402407 34451948

[78] Shi R , Jia S , Liu H , Nie H . Clinical grade lentiviral vector purification and quality control requirements. J Sep Sci. 2022;45:2093‐2101. 35247228 10.1002/jssc.202100937

[79] Mi X , Fuks P , Wang S c , Winters MA , Carta G . Protein adsorption on core–shell particles: comparison of Capto™ core 400 and 700 resins. J Chromatogr A. 2021;1651:462314. 34144396 10.1016/j.chroma.2021.462314

[80] Boi C , Malavasi A , Carbonell RG , Gilleskie G . A direct comparison between membrane adsorber and packed column chromatography performance. J Chromatogr A. 2020;1612:460629. 31668416 10.1016/j.chroma.2019.460629

[81] Fang YM , Lin DQ , Yao SJ . Review on biomimetic affinity chromatography with short peptide ligands and its application to protein purification. J Chromatogr A. 2018;1571:1‐15. 30097342 10.1016/j.chroma.2018.07.082

[82] Lalli E , Silva JS , Boi C , Sarti GC . Membranes affinity membranes and monoliths for protein purification. Membranes. 2019;10:1. 31878114 10.3390/membranes10010001 PMC7022333

[83] Fortuna AR , Taft F , Villain L , Wolff MW , Reichl U . Continuous purification of influenza A virus particles using pseudo‐affinity membrane chromatography. J Biotechnol. 2021;342:139‐148. 34678401 10.1016/j.jbiotec.2021.10.003

[84] Wu, Yuxuan, Eduardo Barbieri, Ryan E. Kilgore, Brandyn D. Moore, Wenning Chu, Gina N. Mollica, Michael A. Daniele, and Stefano Menegatti. “Peptide ligands for the affinity purification of adenovirus from HEK293 and vero cell lysates.” Journal of Chromatography A 1736 (2024): 465396. 10.1016/j.chroma.2024.465396 39342729

