# Supplementary material for: Developing a process of lentivirus purification from recombinant fluids using peptide affinity ligands
Source: Bioeng Transl Med. 2025 Apr 7;10(6):e70017. doi: 10.1002/btm2.70017 (PMC12617543; doi:10.1002/btm2.70017)
Supplement: Supplementary file 1 — APPENDIX S1: Supporting information. [file BTM2-10-e70017-s001.docx]

**Supplementary Information**

## Developing a process of Lentivirus purification from recombinant fluids using peptide affinity ligands

Eduardo Barbieri^1,2^, Gina N. Mollica^1^, Sobhana A. Sripada^1^, Shrirarjun Shastry^1^, Yuxuan Wu^1^, Arianna Minzoni^1^, Will Smith^1^, Elena Wuestenhagen^4^, Annika Aldinger^4^, Heiner Graalfs^4^, Michael S. Crapanzano^2^, Oliver Rammo^4^, Michael M. Schulte^4^, Michael A. Daniele^5,6^, and Stefano Menegatti^1,2,3,6*^

^1^ Department of Chemical and Biomolecular Engineering, North Carolina State University, 911 Partners Way, Raleigh, NC 27606, USA

^2^ LigaTrap Technologies LLC, Raleigh, NC 27606

^3^ Biomanufacturing Training and Education Center (BTEC), North Carolina State University, 850 Oval Dr, Raleigh, NC 27606, USA

^4^ Merck KGaA, Frankfurter Strasse 250, 64293 Darmstadt, Germany

^5^ Joint Department of Biomedical Engineering, North Carolina State University and University of North Carolina at Chapel Hill, 911 Oval Drive, Raleigh, NC 27695, USA

^6^ North Carolina Viral Vector Initiative in Research and Learning (NC-VVIRAL), North Carolina State University, 911 Oval Dr, Raleigh, NC 27695, USA

*^*^ Corresponding author:* [smenega@ncsu.edu](about:blank)*.*

#### S1. Optimizing LVV expression in suspension HEK293F cell cultures

Lentiviruses are almost ubiquitously produced by transfecting HEK293F cells using four plasmids: one carrying the gene of interest (GOI), an envelope plasmid, and two packing plasmids.^71^ While LVVs can be produced in both adherent or suspended HEK293F cells, production in suspension is preferred owing to its easier scalability, higher titers, and no need of supplementation with FBS. Inspired by studies on the effect of cell culture medium composition on LVV and HCP titers,^72,73^ we tested three cell culture media, namely Peak Expression, BalanCD HEK293, and LV-Max. The values of LVV titer measured by transduction assay and HCP titer measured by ELISA are collated in **Figure S1**. When encapsidating a small GOI encoding for green fluorescent protein (GFP, ~ 4.5 kb), no significant difference in LVV titer was observed among the different media; however, the HCP levels in Peak Expression and LV-max media were respectively 1.6- and 2.1-fold higher than those obtained with BalanCD medium (**Figure S1A**). This was anticipated, given the role of medium composition in determining cell metabolism and thus HCP production.^74^

We also investigated the effect of gene payload size on LVV production, by comparing the GOI encoding for GFP (4.5 kb) with one encoding for GFP-fused CRISPR Cas9 (9.5 kb). The results summarized in **Figure S1B** indicate comparable trends, where BalanCD yielded a higher productivity and purity than Peak Expression and LV-Max. However, the functional titer of Cas9-encapsidating LVVs was ~100-fold lower than their GFP-loaded counterparts. These results match the conclusions of Kumar *et al.*, who investigated the effect of GOI size on LVV titers and observed a similar reduction in functional titer as the transgene size increased. ^49^ The authors presented two hypothesis for titer reduction: (*i*) larger GOIs lead to a more challenging encapsulation during viral particle assembly and the transport rate of proviral RNA from the cell nucleus to the cytoplasm decreases with its size.^49^


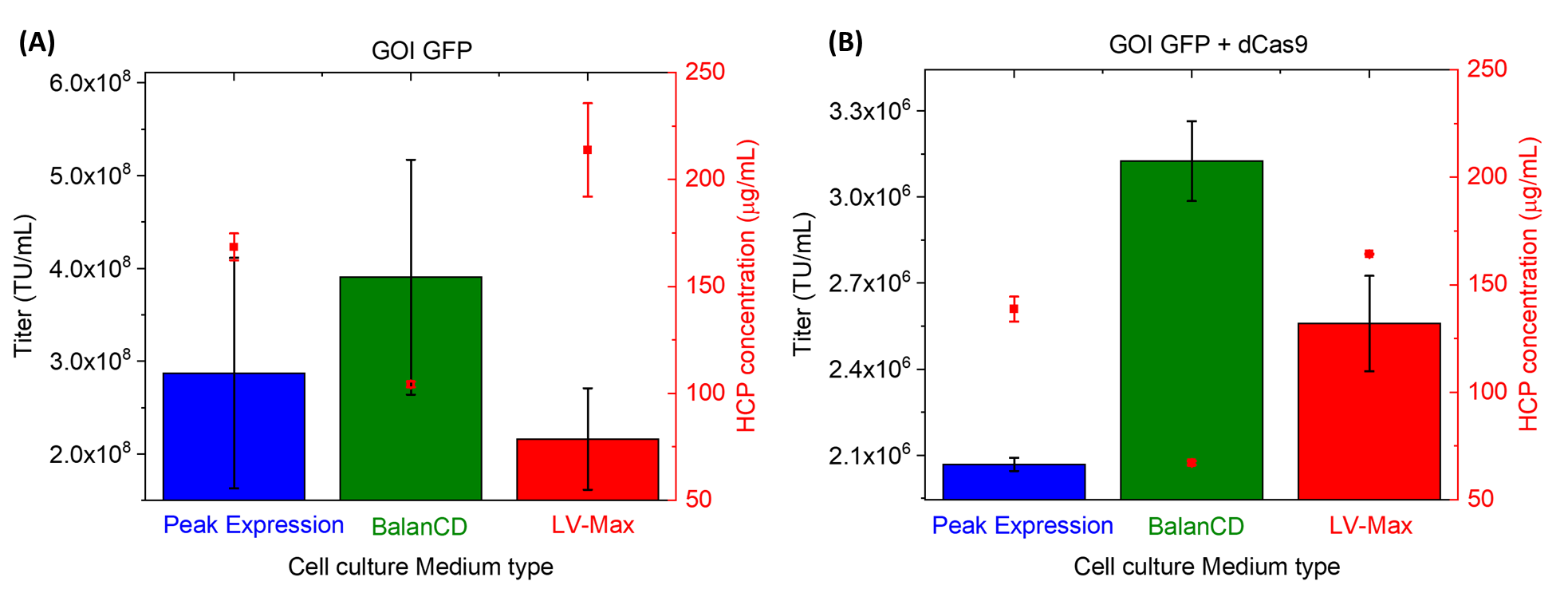


***Figure S1****. Titer of transducing LVV particles (TU/mL) measured by transduction assay (histograms) and HCP titer (red dots) measured via ELISA obtained via transient transfection of HEK293F cells cultured in three cell culture media – namely Peak Expression, BalanCD, and LV-Max – using GOI plasmids* ***(A)*** *pALD-LentiEGFP-K and* ***(B)*** *dCAS9-VP64-GFP.*

Transient transfection, the commonly used method for viral production, is mediated by a transfection reagent that complexes with the plasmids and shuttles them into the host cells. Different transfection reagents have been described in the literature, including calcium phosphate^75^ cationic polymers such as polyethyleneimine (PEI),^76^ and lipids.^77^ To evaluate the role of transfection reagents on functional titer we tested three commercial products, namely TransIT, PEIpro, and LV-Max. The results, summarized in **Figure S2**, indicate that TransIT and LV-Max afforded 3-fold higher titers in comparison to PEIpro. Although the exact composition of these transfection reagents is undisclosed, PEIpro is reportedly a pure cationic polymer while TransIT and LV-Max comprise a mixture of cationic polymers and cationic lipids.

***Figure S2.*** *Comparison of three transfection reagents – namely, TransIT, PEIpro, and LV-Max transfection – for producing LVVs via transient transfection of plasmids pALD-LentiEGFP-K, pALD-Rev-K, pALD-VSV-G-K, and pALD-GagPol-K in HEK293F cells cultured in BalanCD media. Cells were transfected with TransIT and LV-Max upon reaching the density of 4·10^6^ cells/mL (viability > 98%), whereas cells were transfected with PEIpro upon reaching the density of 2.5·10^6^ cells/mL (viability > 98%). The plasmid:transfection reagent ratio (m/v) and total plasmid concentration per mL of cell culture were* ***(A)*** *1:3 and 1 µg/mL;* ***(B)*** *1:1.85 and 1.6 µg/mL;* ***(C)*** *1:3 and 3 µg/mL;* ***(D)*** *1:3 and 1.6 µg/mL;* ***(E)*** *1:3 and 2.5 µg/mL;* ***(F)*** *1:1 and 2.5 µg/mL; and* ***(G)*** *1:2.5 and 2.5 µg/mL.*

***Fgure S3.*** *Structures of* ***(A)*** *GKEAAFAA-,* ***(B)*** *GKEAAFAA-G-,* ***(C)*** *GKEAAFAA-GSG-,* ***(D)*** *GKEAAFAA-GSGPGSG-,* ***(E)*** *GKEAAFAA-GSGSGSG-, and* ***(F)*** *GKEAAFAA-PEG_3_- ligands. The spatial coordinates of the peptide segment (red) in each ligand-spacer construct are fixed to compare the structure and orientation of the spacer arms (A) - (E); the bond tethering the ligand-spacer construct to the adsorbent is marked in green.*

**(A) (B)**

***
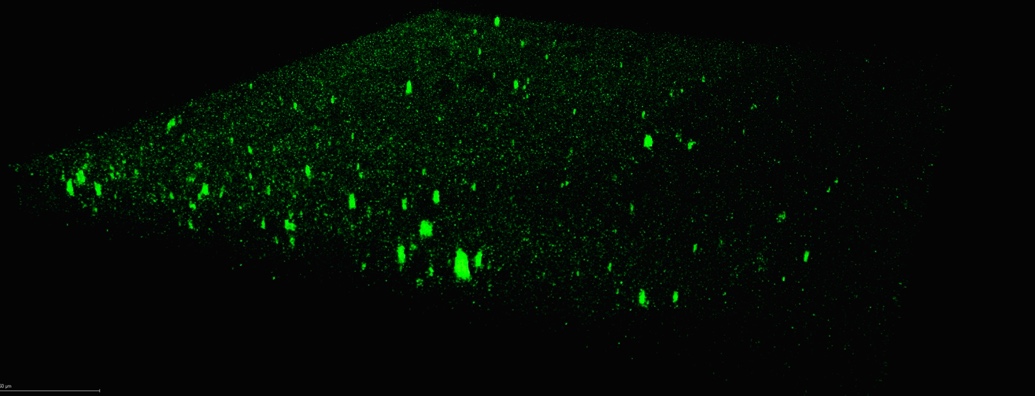
***
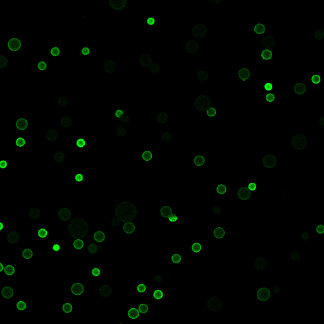


***Figure S4.*** *Confocal images of* ***(A)*** *GKEAAFAA-Poros^TM^ beads and* ***(B)*** *GKEAAFAA-cellulose membrane loaded with pure LVV particles labeled with Syto 13 green fluorescent dye.*

***Table S1.*** *Primer and probe sequences for LVV quantification via real time quantitative pCR (RT-qPCR).*

| Primer | DNA sequence |
| --- | --- |
| Forward primer | CCCAGTTCCGCCCATTCTC |
| Reverse primer | GCCTCGGCCTCTGCATAAATAAA |
| Probe | ATGGCTGACTAATTTTT |

***Table S2.*** *Properties and performance of chromatography resins functionalized with peptide ligand GKEAAFAA. The resins were packed in 1 mL column, equilibrated with 100 mM NaCl in 50 mM PIPES buffer at pH 7.4, and loaded with clarified HEK293F cell culture fluid (LVV titer: 5-9·10^9^ TU/mL; HCP titer: 0.05 mg/mL) at the residence time (RT) of 1 min; following washing, LVV elution was conducted using 0.650 M NaCl in 50 mM PIPES at pH 7.4 at RT of 1 min. Productivity was calculated as the number of cell-transducing LVV units purified by 1 mL of resin in 1 min.*

| **Ligand** | **Resin** | **Polymer** | **Bead Size**  **(µm)** | **Pore Diameter**  **(nm)** | **DBC_10%_**  **(TU/mL of resin)** | **Recovery of**  **Transducing LVVs** | **Productivity**  **(TU/mL·min)** | **HCP LRV** |
| --- | --- | --- | --- | --- | --- | --- | --- | --- |
| GKEAAFAA | ToyoPearl® 650 | PMMA | 65 | 100 | 4.3·10^9^ | 60% | 2.6·10^9^ | 2.07 |
|  | ToyoPearl® 750 |  | 75 | >100 | 5.0·10^9^ | 48% | 2.4·10^9^ | 1.89 |
|  | Eshmuno® 50 µm | PVE | 50 | 50 | 2.2·10^9^ | 49% | 1.1·10^9^ | 2.09 |
|  | Eshmuno® 80 µm |  | 50 | 80 | 1.9·10^9^ | 61% | 1.2·10^9^ | 2.01 |
|  | Genscript iodoacetyl | Agarose | 90 | 130 | 5.6·10^8^ | 71% | 4.0·10^8^ | 2.24 |
|  | WorkBead |  | 45 | 40 | 3.6·10^8^ | 49% | 1.8·10^8^ | 2.24 |
|  | SulfoLink |  | NA | NA | 2.9·10^8^ | 56% | 1.6·10^8^ | 2.09 |
|  | Ultralink | PA/azlactone | 60 | NA | 4.3·10^8^ | 55% | 2.4·10^8^ | 1.75 |
|  | **Poros^TM^** | **PS** | **50** | **50-1000** | **4.5·10^9^** | **65%** | **2.9·10^9^** | **2.04** |
| Camelid  Antibody (V_H_H) | **CaptureSelect™ Lenti**  **VSVG Affinity Resin** | **Agarose** | **65** | **NA** | **2.3·10^8^** | **58%** | **0.7·10^8^**  **(RT: 2 min)** | **2.12** |

*PS: poly(styrene), PMMA: poly(methyl methacrylate), PVE: poly(vinylether), PAM: polyacrylamide.*
